# PARP inhibitor synthetic lethality in ATM biallelic mutant cancer cell lines is associated with BRCA1/2 and RAD51 downregulation

**DOI:** 10.3389/fonc.2024.1380633

**Published:** 2024-05-14

**Authors:** Asli Muvaffak, Kevin G. Coleman

**Affiliations:** Oncology, GlaxoSmithKline, Cambridge, MA, United States

**Keywords:** ATM kinase, DNA double strand break (DSB) repair, homologous recombination deficiency, PARP, synthetic lethality, non-small cell lung carcinoma (NSCLC), genomic instability

## Abstract

**Background:**

Ataxia telangiectasia-mutated (ATM) kinase is a central regulator of the DNA damage response (DDR) signaling pathway, and its function is critical for the maintenance of genomic stability in cells that coordinate a network of cellular processes, including DNA replication, DNA repair, and cell cycle progression. ATM is frequently mutated in human cancers, and approximately 3% of lung cancers have biallelic mutations in ATM, i.e., including 3.5% of lung adenocarcinomas (LUAD) and 1.4% of lung squamous cell carcinomas (LUSC).

**Methods:**

We investigated the potential of targeting the DDR pathway in lung cancer as a potential therapeutic approach. In this context, we examined whether ATM loss is synthetically lethal with niraparib monotherapy. This exploration involved the use of *hATM* knockout (KO) isogenic cell lines containing *hATM* homozygous (-/-) and heterozygous (+/-) generated via CRISPR/Cas9 gene knockout technology in DLD-1, a human colorectal adenocarcinoma cell line. Subsequently, we extended our investigation to non-small cell lung cancer (NSCLC) patient derived xenograft (PDX) models for further validation of poly ADP-ribose polymerase inhibitor (PARPi) synthetic lethality in ATM mutant NSCLC models.

**Results:**

Here, we demonstared that biallelic *hATM* deletion (-/-) in DLD-1 impairs homologous recombination (HR) repair function and sensitizes cells to the PARPi, niraparib. Niraparib also caused significant tumor regression in one-third of the NSCLC PDX models harboring deleterious biallelic ATM mutations. Loss of *hATM* (−/−) was concomitantly associated with low BRCA1 and BRCA2 protein expression in both the *hATM* (−/−) DLD-1 cell line and PARPi-sensitive ATM mutant NSCLC PDX models, suggesting a downstream effect on the impairment of HR-mediated DNA checkpoint signaling. Further analysis revealed that loss of ATM led to inhibition of phosphorylation of MRN (Mre11-Rad50-NBS1) complex proteins, which are required for ATM-mediated downstream phosphorylation of p53, BRCA1, and CHK2.

**Conclusions:**

Taken together, our findings highlight that the synthetic lethality of niraparib in ATM-deficient tumors can be regulated through a subsequent effect on the modulation of BRCA1/2 expression and its effect on HR function.

## 1 Introduction

**Figure 1:**
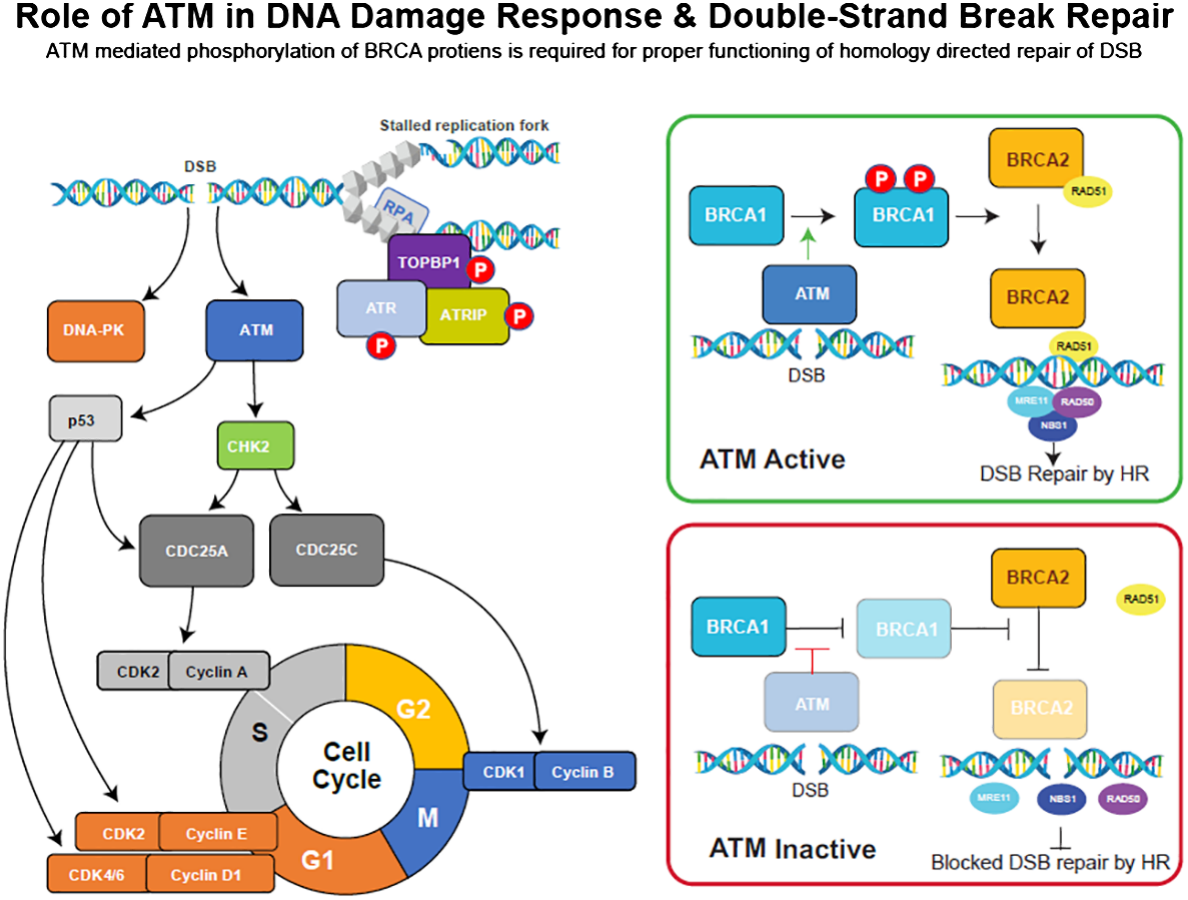
DNA damage repair (DDR) pathway overview and the role of key checkpoint regulators, ATM, ATR, and DNA-PK, in DSB repair processes. Adopted from (11, 12).

DNA damage response (DDR) pathways maintain genome integrity and are activated by errors that occur during DNA replication or by exposure to external agents such as cytotoxic agents or radiation (1). Double-strand breaks (DSBs), which are the most lethal type of DNA lesions, are repaired by homologous recombination repair (HRR), a high-fidelity and efficient DDR pathway that involves multiple interacting proteins, such as BReast CAncer gene 1/2 (BRCA 1/2), ATM, ataxia telangiectasia and rad3-related (ATR) (2, 3), the MRE11/RAD51/NBS1 (MRN) complex (4), and RAD51 (5). The tumor suppressor genes *BRCA1* and *BRCA2* play an essential role in the repair of DSB in the HRR pathway (6), and mutations in either of these genes are known to increase susceptibility to heritable breast, ovarian, pancreatic, and prostate cancers (7–10). In response to DSBs, ATM protein kinase phosphorylates and activates major DNA damage checkpoints, such as p53, serine/threonine checkpoint kinase 2 (CHK2), and BRCA1, which are critical for mediating the effects of ATM on DNA repair (**Figure 1**; 13). ATM also phosphorylates CHK1 (serine/threonine checkpoint kinase 1) in response to ionizing radiation and regulates the G2/M cell cycle checkpoint, preventing cells from undergoing mitosis in the presence of DNA damage (14).

Inherited mutations in *ATM* can result in ataxia-telangiectasia (A-T), a rare genomic instability disease characterized by neurodegeneration, immunodeficiency, sensitivity to radiation, and an increased risk of developing certain malignancies (13). In mice, ATM-deficient embryonic fibroblasts showed deficiencies in G1/S cell cycle checkpoint control following DNA damage, resulting in early senescence (15). Like ATM, ATR phosphorylates multiple substrates in response to DSBs. Additionally, a study revealed that ATR inhibition enhances the anti-tumor activity of cisplatin in ATM-deficient tumor cells (3). The MRN complex (**Figure 1**) is a group of mediator proteins that plays a critical role in ATM signaling, and its loss results in the inability to repair 10%–25% of DSBs (4). RAD51, along with BRCA2, protects stalled replication forks from MRE11-dependent degradation and is critical for DNA replication and HRR (16).

PARP 1/2 enzymes play a key role in the repair of single-strand breaks (SSBs), which are the most common type of DNA lesions. Inhibition of PARP 1/2 results in persistent, unrepaired SSBs, which results in collapsed replication forks and DSB formation. The inability of HR (homologous recombination)-deficient cells to repair DSBs results in the accumulation of mutations and an increased susceptibility to genome instability. The seminal discovery of the synthetic lethality of HR deficiency and PARP inhibition (6, 17) directly led to the development of clinical PARP inhibitors, providing breakthrough therapies for patients with deleterious mutations in *BRCA1/2* and other HRR genes (18–20). In HR-deficient cancer cells, PARPi induces synthetic lethality due to the accumulation of DSBs and PARP trapping, leading to sustained stalling of replication forks and cell death. In addition to BRCA1 and BRCA2, many other interacting genes are involved in the HR pathway and play critical roles in DNA DSB repair (1, 2). While BRCA1 and BRCA2 account for the majority of HRR mutations found in ovarian and breast cancer, deleterious mutations in other non-BRCA HRR genes are found in most cancer types and have been shown to confer sensitivity to PARP inhibitors (21–26). More specifically, *ATM* has been shown to be mutated in many cancers, including lung, gastric, prostate, and mantle cell lymphomas (23, 27–31). Approximately 3% of lung cancers harbor mutations in the *ATM* gene, i.e., 3.5% of adenocarcinomas and 1.4% of squamous cell carcinomas (32, 33). Moreover, loss of ATM protein expression has been reported in over 40% of lung adenocarcinomas (28). Importantly, loss of ATM has been shown to increase PARPi sensitivity in lung, prostate, and colorectal cancers (13, 22, 30, 34). Although ATM is known to play an important role in DSB repair, the mechanism by which cells respond to PARPi treatment in the absence of ATM is not fully understood.

NSCLC accounts for approximately 86% of all lung cancer diagnoses (35) and is the most frequent cause of cancer-related mortality worldwide (36). Existing therapies for NSCLC do not benefit all patients. Targeted therapies against anaplastic lymphoma kinase (ALK) fusion and epidermal growth factor receptor (EGFR) mutations are effective in <20% of NSCLC patients whose tumors harbor these mutations (37). Even immunotherapy approaches such as PD-1:PD-L1 blockade, which are more widely efficacious than ALK and EGFR inhibitors, do not benefit a substantial proportion of patients with NSCLC (3, 13, 38, 39).

ATM is the most frequently mutated DNA damage repair gene in NSCLC (approximately 3% of samples), and is significantly associated with mutations in KRAS, but mutually exclusive with EGFR mutations (40). These observations suggest a potential opportunity for the PARP1-selective inhibitor niraparib to serve as an effective treatment for NSCLC patients with ATM mutations who cannot benefit from EGFR-targeted therapies. Niraparib received FDA-approval as a maintenance monotherapy treatment of ovarian cancer, fallopian tube cancer, and peritoneal cancer (19) and as a combination partner with abiraterone for the treatment of prostate cancer (41). Notably, in a Phase I trial, niraparib monotherapy was associated with antitumor activity in two patients with NSCLC (42). While the ATM mutational status of these patients was not disclosed, this observation aligns with preclinical studies indicating that the loss or downregulation of ATM is synthetic lethal with PARP inhibitors in gastric and colorectal cancer cell lines (43, 44). Moreover, deletion of ATM in mouse models of lung cancer and pancreatic cancer induced sensitivity to PARP inhibitors, as observed in studies by Schmitt et al. (29) and Perkhofer et al. (34).

To investigate the potential of using PARP inhibitors to target the DDR pathway in lung cancer as a novel therapeutic approach, *hATM* KO isogenic cell lines were used to help elucidate the molecular mechanism associated with the synthetic lethality between *hATM* KO and PARPi treatment. Due to our inability to identify a lung cancer cell line exhibiting tolerance to the loss of BRCA2, we sought to incorporate both *hATM* (−/−) and *hBRCA2* (−/−) isogenic cell lines into our studies. We elected to use the DLD-1 colorectal adenocarcinoma cell line to generate the *hATM* KO cell line because we were unable to identify a lung cancer cell line that tolerated the loss of BRCA2 and it had previously been established that DLD-1 cells tolerate the homozygous loss of BRCA2 (6, 45). To increase the clinical relevance of our findings, we also utilized ATM mutant PDX models to assess the potential efficacy of niraparib in treating NSCLC patients with ATM mutations. Our findings indicated that ATM loss is synthetically lethal with niraparib monotherapy in both ATM KO cell lines and NSCLC PDX models. We found that homozygous loss of *ATM* caused a marked increase in the *in vitro* and *in vivo* sensitivity of tumor cells to niraparib, which is consistent with previous findings (29, 34, 46, 47). Furthermore, we demonstrated that loss of ATM protein was associated with decreased levels of BRCA1 and BRCA2, suggesting downstream impairment of HR-mediated DNA checkpoint signaling. Loss of ATM also led to the inhibition of phosphorylation of MRN-complex proteins, which are required for ATM activation and downstream phosphorylation of p53, BRCA1, and CHK2. Our results highlight that the PARPi synthetic lethality phenotype observed in ATM-deficient cancer cells may be regulated through the subsequent loss of BRCA1/2. Taken together, these findings suggest that defects in HRR sensitize lung tumors to niraparib, and loss of ATM contributes to PARPi efficacy in NSCLC tumors. Our observations are important because they support the potential of targeting the DDR pathway as an alternative therapeutic approach for NSCLC patients.

## 2 Materials and methods

### 2.1 Cell lines and niraparib treatment conditions

The DLD-1 parental cell line was obtained from the ATCC (CCL-221). The cell line authentication details are listed in the **Supplementary Data**. All cell lines were grown in RPMI-1640 medium supplemented with 10% fetal bovine serum (Atlanta Biologicals Inc., GA), as recommended by ATCC. All cultures were maintained at 37°C in a humidified 5% CO_2_ atmosphere and tested periodically to ensure the absence of mycoplasma. Generally, cells are passaged upon reaching ~75% confluency.

Tool inhibitor stocks and dilutions were prepared in dimethyl sulfoxide (DMSO). The PARP inhibitor, niraparib (Zejula/MK-4827), was obtained from Tesaro/GSK.

### 2.2 Generation of CRISPR/Cas9 KO cell lines

Applied StemCell (ASC, a QHP company) used their proprietary CRISPR-Cas9 technology to introduce Cas9/guide RNA (gRNA) complexes into the target cells, DLD-1. Cas9/gRNA-mediated indel formations at the targeted regions caused by non-homologous end joining (NHEJ) result in frameshift and/or premature stop, thus generating a knockout of the gene of interest. Dual sgRNA approach was utilized to generate a homozygous and a heterozygous KO of *hATM* or *hBRCA2* in DLD-1 cell line (**Table 1**; **Supplementary Table S1**). Briefly, after transfecting gRNA and Cas9, single cell-derived clones were screened and identified for homozygous KO clones, heterozygous KO clones, and isogenic wildtype clones. CRISPR/Cas9 was prepared by transfecting the corresponding plasmid DNAs into DLD-1 cells using Lipofectamine 2000 (Invitrogen, 52887) in OptiMEM (GIBCO, 31985-070) media.

**Table 1:** List of DLD-1 ATM and BRCA2 KO cell lines with genetic alteration.

	Clone/Cell Line ID	Clone Description (bp alteration/allele/sgRNA sequence)	Genetic Modification	Vector backbone
1	Dld-1_hATM KO_HOMO_A7	hATM Homozygous KO (+1bp)	CRISPR/Cas9 KO	pX330-U6-Chimeric_BB-CBh-hSPCas9 co-expression vector
2	Dld-1_hATM KO_HET_D4	hATM Heterozygous KO (WT, −16bp)	CRISPR/Cas9 KO	pX330-U6-Chimeric_BB-CBh-hSPCas9 co-expression vector
3	Dld-1_hBRCA2 KO_HOMO_2B4	hBRCA2 Homozygous KO (−10bp, −1bp)	CRISPR/Cas9 KO	pX330-U6-Chimeric_BB-CBh-hSPCas9 co-expression vector

#### 2.2.1 Screening of positive clones carrying the desired KO

Genomic DNA from each single-cell-derived clone was extracted, and PCR was performed to amplify the targeted region. Next-generation sequencing (NGS) or Sanger sequencing was performed to sequence the PCR products and identify desired clones. The level of knockout of ATM and BRCA2 proteins in DLD-1 *hATM* homozygous KO (−/−), *hATM* heterozygous (+/−) KO, *hBRCA2* homozygous KO (−/−) clone 2B4 (internally generated), and *hBRCA2* heterozygous (+/−) KO in individual KO clones were determined by WB analysis, and NGS analysis confirmed the corresponding gene edits in heterozygous and homozygous clones. A commercially available DLD-1 cell line containing homozygous (−/−) KO of *hBRCA2* was also purchased from Horizon Discovery (Cat no. HD 105-007).

### 2.3 Cell proliferation assays

#### 2.3.1 Colony formation assay

##### 2.3.1.1 Cell culture

DLD-1 cell lines were cultured in RPMI 1640 medium Glutamax (Thermo Fisher, Cat. no. 61870), supplemented with heat-inactivated fetal bovine serum (FBS, Thermo Fisher, Cat. no. 10500), 25 mM Hepes (ThermoFisher, cat. no. 15630), and 50 μg/mL Gentamicin (ThermoFisher, cat. no. 15750060). Cells were maintained at 37°C, 5% CO2 and split every 3 or 4 days.

##### 2.3.1.2 Colony formation assay

Upon detachment from flasks, cells were counted using the ViCell XR and suspended to desired concentrations by serial dilutions. A volume of 500 μL of cell suspension were seeded in tissue culture 24-well plates (Perkin Elmer, Cat. no. 1450-605) and treated with 7-point dose titrations of niraparib, that is, 50 μM to 50 pM (1:10 dilution), and vehicle (DMSO) were incubated at 37°C and 5% CO_2_ for 14 days. For concentration response curves, compounds were diluted in DMSO and added to cells 1 day after seeding. Media containing the compounds were replaced every 3–4 days/week. Cells were cultured for 14 days, and the medium with compounds was changed every 3 or 4 days. At experimental endpoint (14 days), the medium was removed from cell cultures, and 200 μL of 10 μg/mL CellMaskTM deep red plasma membrane stain (Thermo Fisher, Cat. no. C10046) prepared in complete medium were added to cells. Samples were incubated at 37°C, 5% CO_2_ for 15 min. After cell labeling, dye was removed from the plate, and 250 μL 4% PFA and 1× PBS were added to cells and incubated for 15 min at room temperature. Fixation was followed by three washes with 300 μL 1× PBS, and image acquisition was performed at IN Cell Analyzer 2200 (GE Healthcare) with a ×2 objective (four images per well were acquired).

A protocol for image analysis was used in Columbus environment on the four fields-stitched images. Such protocol included image processing and colony selection. For image processing, background subtraction was performed on the Cy5 channel by applying the “Sliding Parabola” preprocessing algorithm, with curvature 10. The “Find Image Region” tool was applied to preprocessed images based on absolute threshold to identify objects with intensity higher than an empirically determined threshold (~500). In the colony selection phase, objects with area below 0.05 mm^2^ were not classified as colonies and excluded from the analysis. The number of resulting objects and total colony area were used as output parameters for the assay.

#### 2.3.2 3D clonogenic assay

3D clonogenic assays with DLD-1 *hBRCA2* (−/−), *hATM* (+/−), and *hATM* (−/−) KO cell lines were performed using ultra-low attachment plates, where cells were seeded in 50 μL cell culture medium containing 0.4% (w/v) soft agar and overlaid with 100 μL culture medium with or without test compounds. Compound addition was performed 24 h after cell seeding, and then, every 3–4 days (two times/week) during the incubation period. After 14 days of incubation, the colonies were stained with 2-(4-iodophenyl)-3-(4-nitrophenyl)-5-phenyltetrazolium chloride and counted using an automated image analysis system (Bioreader 5000 V-alpha, BIO-SYS GmbH). Sigmoidal dose–response curves were fitted to the data points (T/C values) obtained for each cell line using a four-parameter non-linear curve fit (Charles River DRS Datawarehouse Software).

In vitro activity was calculated using the following formula: CGI% (colony growth inhibition %): [1−(TIC50/CIC50)]×100, where TIC50 = niraparib IC50 on DLD-1 *hATM* KO (+/−), (−/−) or *hBRCA2* (−/−) KO and CIC50 = niraparib IC50 on DLD-1 parental cell line.

#### 2.3.3 Data analysis for colony formation assay

Readouts used in the automated image analysis were number of colonies per well and total colony area:


$$Total\;colony\;area\;\left[\%\right]=\frac{Sum\;of\;the\;area\;occupied\;by\;colonies}{Area\;of\;the\;well}\times 100$$

To normalize results and compare different cell lines, data are reported as surviving fraction:


$$Surviving\;fraction\;\left[\%\right]=\frac{nr\;of\;colonies}{nrof\;colonies\;of\;vehicle}\times 100\;Surviving\;fraction\;\left[\%\right]=\frac{total\;colony\;area}{total\;colony\;area\;of\;vehicle}\times 100$$

All data were collected in one excel file and then plotted and analyzed using GraphPad statistical software. Four parametric logistic equation is used for data fitting:


$$Y=\frac{Bottom-\left(Top-Bottom\right)}{1+10^{\left(log\;1C_{50}-X\right)\times HillSlope}}$$

where Y was the assay response, X the log compound concentration, Top and Bottom the maximal and minimal signal at plateau, and IC50 the compound concentration inhibiting 50% of the maximal effect. If the bottom or top of the curve were not well defined by the plateau, fitting was constrained using maximal compound concentration and vehicle treatments as minimal and maximal cell growth, respectively. Statistical analysis between pIC50 values was performed using unpaired t-test.

#### 2.3.4 Niraparib single-agent IC50 dose–response curves

For IC50 calculations, these data were fitted with a standard three-parameter dose–response non-linear regression fit using the GraphPad Prism software.

In vitro activity was calculated using the following formula: CGI% (colony growth inhibition %): [1−(TIC50/CIC50)]×100, where TIC50 = niraparib IC50 on DLD-1 *hATM* KO or *hBRCA2* KO and CIC50 = niraparib IC50 on DLD-1 parental cell line.

### 2.4 High content immunofluorescence γH2AX, geminin, and Rad51 foci imaging assay

The homologous recombination (HR) capacity for DNA double-strand break repair was assessed using a high content immunofluorescence imaging assay (Horizon Discovery) to quantify nuclear RAD51 and γH2AX-foci in *hBRCA2*- and *hATM*-deficient DLD-1 cell lines after inducing DNA SSBs following hydrogen peroxide treatment and 3 µM niraparib treatment for 18 h.

### 2.5 Staining procedure

All plates were stained simultaneously. For each time point, there were two plates: Plate 1, γH2A.X, and Plate 2, Geminin and Rad51. The following steps were performed at room temperature unless otherwise specified. Cells were washed once in PBS for 10 min and permeabilized with PBS/0.1% Triton X-100 for 2 min. Cells were blocked with 10% goat serum (CST) in PBS/0.1% Triton-X-100 for 1 h. Cells were washed three times for 10 min per wash with PBS/0.1% Triton-X-100.

#### 2.5.1 γH2A.X staining

Cells was incubated withγH2A.X antibody (1:2,000 dilution, Merck Millipore #05-636 LOT 3076468) in PBS/0.1% Triton-X-100 for 1 h. Cells were washed three times for 10 min per wash with PBS/0.1% Triton-X-100. Cells were incubated with goat anti-mouse IgG (H+L) Cross-Absorbed Alexa Fluor 488 (1:500 dilution, Thermo Fisher, A-11001) and Hoechst 33342 (1 μg/ml) in PBS/0.1% Triton-X-100 for 1 h. Cells were washed a final three times for 10 min per wash in PBS/0.1% Triton X-100 before wells were filled with PBS, the assay plates were sealed and stored at 4°C until imaging.

#### 2.5.2 Geminin and RAD51 staining

Cells were incubated with RAD51 antibody (1:1,000 dilution, Abcam, ab133534, Lot: GR219215-35) in PBS/0.1% Triton-X-100 at 4°C overnight. Cells were washed three times for 10 min per wash with PBS/0.1% Triton-X-100. Cells were incubated with Geminin antibody (1:250 dilution, Santa Cruz, (H-3): sc-374187, Lot 11311 in PBS/0.1% Triton-X-100 for 1 h. Cells were washed a further three times for 10 min per wash with PBS/0.1% Triton-X-100 before being incubated with goat anti-mouse IgG (H+L) Cross-Absorbed Alexa Fluor 488 (1:500 dilution, Thermo Fisher, A-11001), goat anti-rabbit IgG (H+L) Cross-Absorbed Alexa Fluor 568 (1:500 dilution, Thermo Fisher, A-11036), and Hoechst 33342 (1 μg/mL) in PBS/0.1% Triton-X-100 for 1 h. Cells were washed a final three times for 10 min per wash in PBS/0.1% Triton X-100 before wells were filled with PBS, the assay plates sealed and stored at 4°C until imaging.

#### 2.5.3 Imaging and Analysis for γH2A.X, Geminin, and RAD51 Assay

Each plate was imaged using a ×20 and 40 objective in an open aperture (widefield) mode on a high content imager (IN Cell Analyzer 6000, GE Healthcare), with 15 fields acquired per well. Images were recorded with the ×20 objective were analyzed using Cell Profiler software (version 2.2.0). The analysis involved four steps: (1) a global threshold was applied to the Hoechst image using a manual threshold of 0.04 (2,621.4/65535). Nuclei were identified as objects 30–175-pixel units in diameter, with a shape form factor of >0.6. Any clumped nuclei were distinguished and separated in shape. Any nuclei touching the border of the image were excluded. For Geminin staining, a mean nuclei Geminin intensity was determined; this was applied to a threshold filter and cells classified as either Geminin positive or negative. Images were background corrected to enhance foci (rolling ball size, 5 pixels). A Laplacian filter (smoothing, 0.25) was applied and local maxima detected in ImageJ. Both background-corrected and local maxima images were imported into Cell Profiler. Local maxima served as seed points for the foci mask. Nuclear foci were identified from the background-corrected images by expansion of those seed points that fell within the nuclear mask and only across those pixels that exceeded background. Manual thresholds were set, and foci were filtered based on size (spot diameter between 3 and 15 pixels). Parameters measured included an object’s size, shape, and intensity.

#### 2.5.4 Processing of images

Images ×40 (332.8 μm × 332.8 μm, each pixel=162.5 nm) were processed in Adobe Photoshop. The two channels were merged, and each channel was manually adjusted using the levels tool in Photoshop and set at 70 for DAPI and 20 RAD51. The same conditions were applied to each image in the collection. A representative field was selected and cropped to a size of 400 × 400 pixels. From this cropped image, each channel was added into a slide deck together with a merger of both channels.

For all results, the mean foci count and the percentage of foci-positive cells (>10 γH2A.X or >5 RAD51 foci/nucleus) were plotted. The average of the duplicate wells + SEM is shown. The percentage of Geminin-positive nuclei was plotted. Only when more than 200 Geminin-positive nuclei were identified was the percentage of RAD51 and Geminin double-positive cells plotted to minimize bias due to small sample size. Assay performance graphs show the response of all cell lines to etoposide treatment. Similarly, graphs were provided that plotted the response of all cell lines to the full niraparib dose range. As increases in foci number only occurred at the four highest niraparib doses, each dose was plotted separately. To allow direct comparison of foci number and percentage of foci positive, all cell lines were plotted together on the same graph for each dose; additionally, the data were plotted separately for each individual cell line.

### 2.6 WB analysis of HR function

RAD51 and γH2AX protein levels in DLD-1 *hBRCA2*(−/−) KO, DLD-1 *hATM* (−/−) KO, and ATM/BAP1/MRE11A/XRCC2/RAD51D mutant NSCLC PDX models were analyzed in vehicle- and niraparib-treated tumors at termination.

#### 2.6.1 Protein detection

Cell lysates for Western blotting were collected using RIPA lysis buffer (Boston Bioproducts, BP-115) supplemented with 1× Halt Protease Inhibitor (VWR International, VWR No. PI78415), 5 mM NaF (Sigma-Aldrich Co.), and 10mM β-glycerol phosphate (Sigma-Aldrich Co.). Proteins (10–15 μg) were separated on 4%–15% Mini-PROTEAN TGX™ Precast Gels (BioRad Laboratories, 456-1086), and on 4%–15% Criterion™ TGX™ Precast Gel (Bio-Rad Laboratories, 5671085). Proteins were transferred onto a PVDF membrane using the TransBlot^®^ Turbo™ Transfer System (Bio-Rad Laboratories, 170-4155).

Protein levels from Western blots were quantified by densitometry using ImageJ software and normalized to the loading control for each sample. The band signal intensities were obtained from raw chemiluminescent images of the films.

#### 2.6.2 Antibodies

Western blotting was performed with antibodies against ATM (Cell Signaling Technologies (CST), D2E2, Rabbit mAb no. 2873), BRCA1 (CST, Rabbit mAb no. 14823), BRCA2 (CST, D9S6V, Rabbit mAb no. 10741), RAD51 (CST, D4B10, Rabbit mAb no. 8875), γH2AX [CST, phospho-histone H2AX (Ser 139), 20E3, Rabbit mAb no. 9718], H2AX (CST, Histone H2AX Antibody no. 2595), pRAD50 (Ser 638, CST, Rabbit Antibody no. 74778), RAD50 (CST, E3I8K, Rabbit mAb no. 86225), pNBN [Ser 343, Phospho-p95/NBS1 (Ser343) Rabbit Antibody no. 3001], p95/NBS1 (CST, D6J5I, Rabbit mAb no. 14956), GAPDH [CST, 14C10, rabbit mAb (HRP conjugate) no. 3683], β-actin (CST, 13E5, Rabbit mAb no. 4970), and BioRad Goat Anti-Rabbit IgG (H+L)-HRP conjugate (no. 170-6515).

### 2.7 In vivo studies in CDX and PDX models

Niraparib monotherapy experiments were performed using female NMRI nude mice (CRL: NMRI-Foxn1nu) delivered at the age of 4–6 weeks, used for implantation after at least 1 week of quarantine. Cell lines bearing a homozygous (−/−) or a heterozygous (+/−) deletion of *hBRCA2* or *hATM* were subcutaneously implanted into mice (5×10 ^6^ cells/mouse). Mice were randomized into different treatment groups (8–12 animals per treatment group) when tumors reached 50–100 mm^3^. The animals were then divided into two groups, with the first group serving as a control and receiving vehicle therapy and the second group receiving niraparib at 50 mg/kg/day. This randomization process ensured a balanced distribution of animals among the experimental groups for a robust assessment of the antitumor efficacy of niraparib. The first group was a control group that received vehicle therapy at 5 ml/kg/day (0.5% methyl cellulose), and the second group was treated with niraparib at 50 mg/kg/day. All treatments were administered daily via oral gavage until the experimental end point was reached. The antitumor efficacy of niraparib was assessed using a vehicle control group as a reference.

Niraparib single-agent activity was evaluated in 10 lung cancer PDX models with biallelic mutations in a set of clinically relevant HRR genes. PDX models were obtained from Charles River Labs, Crown Bioscience, and Champions Oncology (**Supplementary Table S3**). Niraparib was administered at 50 mg/kg dose over a period of 28 days or longer orally, once daily. Tumor growth was monitored twice per week.

Tumor growth inhibition was determined by comparing the relative tumor volume (RTV) of the niraparib-treated group with those of the vehicle control group. Tumor growth inhibition was expressed as T/C_50_, which is the T/C value on the last day on which at least 50% of animals remained in the respective groups in percent. Individual RTVs of the test and control groups were compared on days the T/C_50_ value was achieved in the test group and evaluated for statistical significance of antitumor efficacy with the non-parametric U-test (Mann–Whitney–Wilcoxon). In vivo activity was assessed using the following formula: tumor growth inhibition% (TGI%): [1−(ΔT/ΔC) × 100. As to response criteria, according to NCI standards, a T/C ≤ 42% is the minimum level of anti-tumor activity, and a T/C <10% is considered a high anti-tumor activity level.

## 3 Results

### 3.1 Homozygous loss of ATM is synthetic lethal with niraparib, in vitro studies

**Figure 2:**
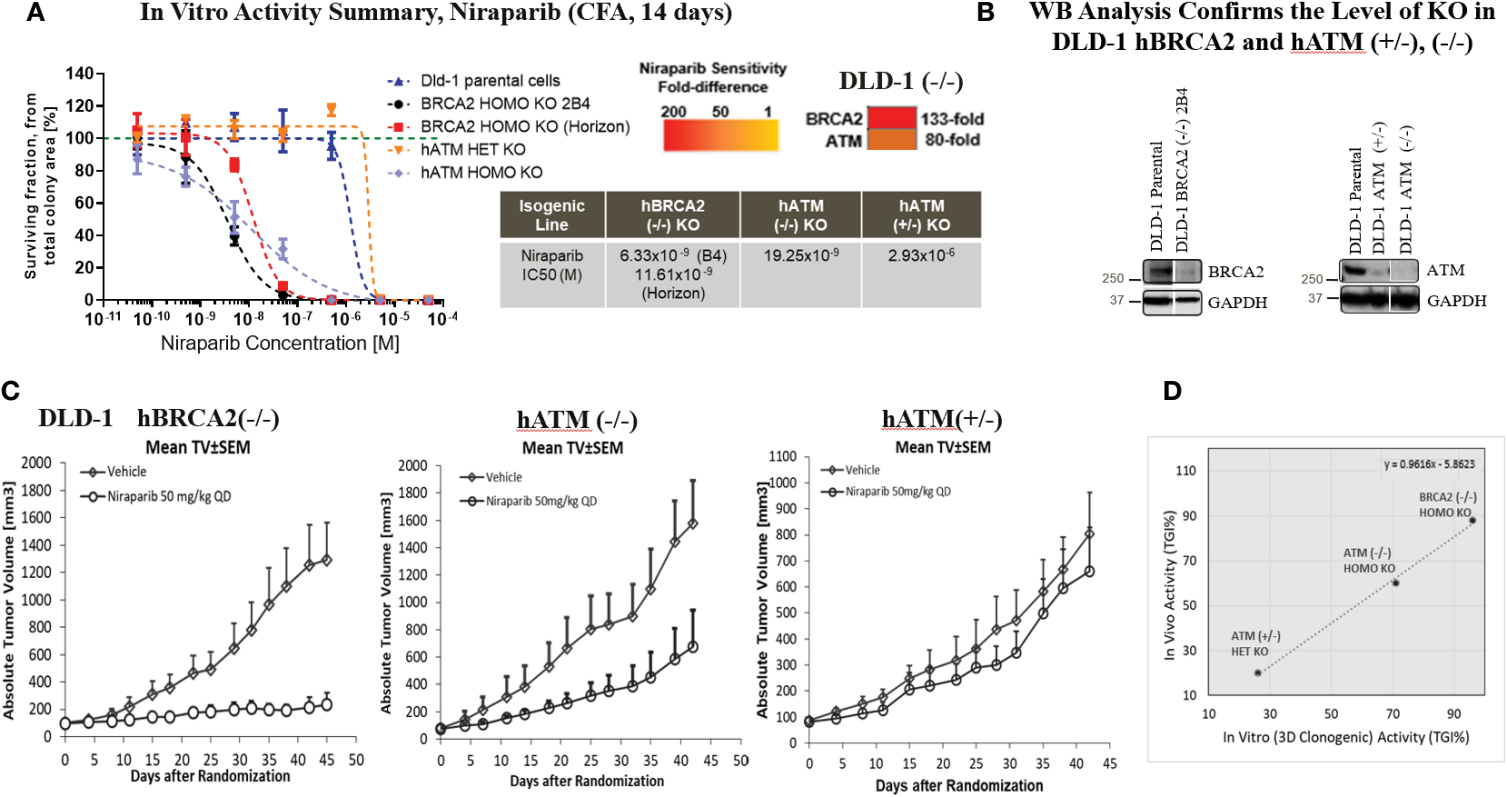
Homozygous loss of *ATM* resulted in a marked increase in the in vitro and in vivo sensitivity of tumor cells to niraparib. Dose response curves for niraparib in *BRCA2* (−/−, clone 2B4, internally generated, and Horizon Discovery, Cat. no. HD 105-007), *hATM* (−/−), and *hATM* (+/−) KO cell lines and niraparib sensitivity summary and sensitivity ranking using 2D colony formation assay (CFA), heatmap summary **(A)**, WB analysis to confirm the level of KO in DLD-1 *hB*RCA2 (−/−) and *hA*TM (−/−) **(B)**, niraparib in vivo activity in DLD-1 *hBRCA2* (−/−, Horizon Discovery), DLD-1 *hATM* (−/−) KO and DLD-1 *hATM* (+/−) KO xenografts **(C)**, and comparison of in vitro vs. in vivo activity of niraparib in DLD-1 *hBRCA2* and *hATM* (+/−), (−/−) KO cell lines from 3D clonogenic assay **(D)**. In vivo activity, tumor growth inhibition% (TGI%): [1−(ΔT/ΔC)]×100; response criteria: according to NCI standards, a T/C ≤ 42% is the minimum level of anti-tumor activity and a T/C <10% is considered a high anti-tumor activity level; in vitro activity (CFA), CGI% (colony growth inhibition %): [1−(TIC50/CIC50)]×100, where TIC50 = niraparib IC50 on DLD-1 *hATM* KO or *hBRCA2* KO and CIC50 = niraparib IC50 on DLD-1 parental.

Given the well-established synthetic lethality between BRCA deficiency and PARP inhibition, our objective was to evaluate PARPi sensitivity in *hATM*-deficient cancer cell lines relative to *hBRCA2*-deficient cells. To this end, we generated isogenic cell lines containing homozygous (−/−) and heterozygous (+/−) knockout (KO) of *hATM* as well as homozygous (-/-) KO of *hBRCA2* using CRISPR/Cas9 gene editing technology in the DLD-1 cell line (**Table 1**; **Figures 2A, B**). In addition, we purchased a commercially available DLD-1 cell line containing homozygous (−/−) KO of *hBRCA2* (Horizon Discovery, Cat. no. HD 105-007) for comparative analysis.

Niraparib treatment of the DLD-1 *hATM* KO (−/−) cell line significantly inhibited colony formation with an IC50 value of 19.25×10^−9^ M compared to an IC50 value of >10×10^−5^M in the DLD-1 parental cell line. This indicated that the biallelic loss of *hATM* resulted in an 80-fold increase in sensitivity to niraparib compared to the HR-proficient DLD-1 parental cell line (**Figure 2A**). The heterozygous loss of *hATM* (+/−) in the DLD-1 cell line did not alter sensitivity to niraparib. In comparison, the DLD-1 *hBRCA2* (−/−) KO clone B4 cell line (internally generated) showed a ~3-fold higher sensitivity to niraparib compared to the cell line with biallelic loss of ATM, with an IC50 value of 6.33×10^−9^ M. Similar results were observed with the externally resourced DLD-1 *hBRCA2* KO (Horizon Discovery, Cat. no. HD 105-007) cell line, i.e., ~2-fold higher sensitivity observed, with an IC50 value of 11.61×10^−9^ M. This observation highlighted a 133-fold increase in sensitivity to niraparib treatment for DLD-1 *hBRCA2* (−/−) clone B4 cell line in comparison to the DLD-1 parental cell line (**Figure 2A**). Similar data were obtained using *hATM* (−/−) and *hBRCA2* (−/−) isogenic HeLa cell lines (**Supplementary Table S2**).

### 3.2 Homozygous loss of ATM is synthetic lethal with niraparib, in vivo studies

The in vitro studies were extended in vivo by investigating the efficacy of niraparib in NMRI nu/nu mice bearing DLD-1 *hBRCA2* (−/−), DLD-1 *hATM* (−/−) KO, and DLD-1 *hATM* (+/−) KO cell lines grown as subcutaneous tumor xenografts. The highest antitumor efficacy was detected in the DLD-1 *hBRCA2* homozygous KO (TGI%, 88%), and significant efficacy was achieved in the DLD-1 *hATM* homozygous KO cell line (TGI, 60%), whereas the DLD-1 *hATM* (+/−) heterozygous KO was relatively insensitive to niraparib treatment at a dose of 50 mg/kg once daily (**Figure 2C**). Importantly, a direct correlation was observed when comparing the in vitro and in vivo potency of niraparib in the three DLD-1 isogenic cell lines. More specifically, the *hBRCA2* (−/−) cell line demonstrated the highest degree of niraparib sensitivity (>90% TGI in vivo, >85% CGI in vitro), with *hATM* (−/−) cell line having the second highest degree of niraparib sensitivity (60% TGI in vivo, ~70% CGI in vitro) and the *hATM* (+/−) cell line being the least sensitive cell line to niraparib (<30% TGI/CGI in both in vivo and in vitro, **Figure 2D**).

### 3.3 Loss of ATM is associated with a disruption of HR-mediated DNA damage response signaling

**Figure 3:**
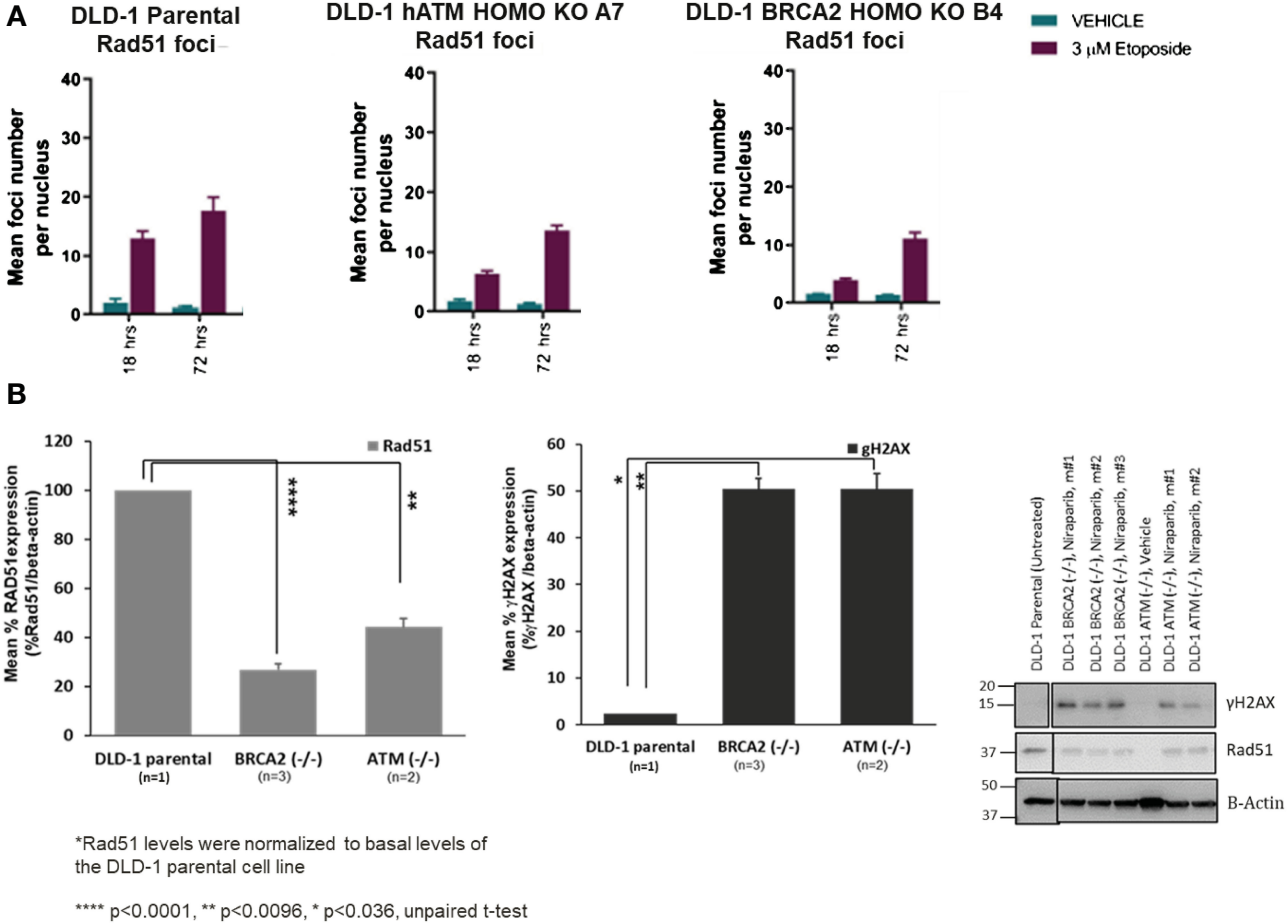
The mean foci distribution of RAD51 (in vitro) at 3 µM of etoposide treatment in DLD-1 parental, DLD-1 *hBRCA2* HOMO KO B4, and DLD-1 *hATM* HOMO KO at 18 h and 72 h was plotted (the mean foci count and the percentage of foci positive cells, i.e., >5 RAD51 foci/nucleus are shown. The average of the duplicate wells + SEM is shown. The percentage of Geminin-positive nuclei was plotted. Only when more than 200 Geminin-positive nuclei were identified was the percentage of RAD51 and Geminin double-positive cells plotted to minimize bias due to small sample size) **(A)**. Changes in γH2AX and Rad51 protein levels in DLD-1 hBRCA2 (−/−) and DLD-1 hATM (−/−) KO, from niraparib treated in vivo tumors at termination **(B)**.

The molecular mechanisms underlying PARPi synthetic lethality in HR-deficient cancer cells beyond BRCA1/2 gene deficiencies have not been fully elucidated. To investigate the impact of ATM loss on DNA repair function, we assessed the homologous recombination repair capacity in the DLD-1 *hATM* (−/−) cell line. Specifically, high-content immunofluorescence-based nuclear foci analysis indicated that the number of RAD51-foci, a surrogate marker of HRR functionality, was approximately two- and four-fold decreased in the DLD-1 *hATM* (−/−) and DLD-1 *hBRCA2* (−/−) cell lines, respectively, compared to the parental DLD-1 cell line following etoposide treatment (at 3 μM for 18–72 h) (**Figure 3A**, **Supplementary Figures S1**-**S3E–G**). The observation that the DLD-1 *hBRCA2* (−/−) cell line has fewer RAD51 foci than the DLD-1 *hATM* (−/−) cell line suggests that BRCA2 loss causes a higher degree of DDR deficiency compared to ATM loss and is consistent with the observation that DLD-1 *hBRCA2* (−/−) cells are approximately three-fold more sensitive to niraparib treatment compared to DLD-1 *hATM* (−/−) cells (**Figures 2A**, **3A**). We also assessed the degree of DNA damage induced by etoposide in the *hATM* (−/−), *hBRCA2* (−/−), and parental DLD-1 isogenic cell lines by determining the number γH2AX foci, a well-established marker of DSBs, in each cell line. The number of γH2AX foci were markedly increased in both the DLD-1 *hATM* (−/−) and *hBRCA2* (−/−) cell lines following etoposide treatment compared to the vehicle treatment for 18 h and 72 h (**Supplementary Figures S1**-**S3A–D**).

### 3.4 Loss of ATM is associated with decreased RAD51 and γH2AX protein expression in vivo

Because of the difficulty of examining RAD51 foci numbers in tumor xenograft tissue samples, we examined the molecular mechanisms underlying niraparib synthetic lethality in *hATM* (−/−) tumor xenografts by examining RAD51 and γH2AX protein expression levels following a prolonged niraparib treatment period (50 mg/kg QD dosing regimen for a period of ~45 days). RAD51 protein expression levels were significantly lower in the DLD-1 *hATM* (−/−) tumor xenografts compared to that in the parental HR-proficient DLD-1 tumor xenograft (**Figure 3B**). Even higher suppression of RAD51 expression was consistently observed in the DLD-1 *hBRCA2* (−/−) xenografts, which is consistent with their greater sensitivity to niraparib compared to the DLD-1 *hATM* (−/−) KO cell line in the in vivo efficacy study (**Figure 2C**). Additionally, although damage-induced formation of γH2AX foci was compromised to similar degrees in the *hATM* (−/−), *hBRCA2* (−/−), and parental DLD-1 cell lines grown in vitro (**Supplementary Figure S3D**), prolonged niraparib treatment caused a marked increase in γH2AX protein levels in the *hATM* (−/−) and *hBRCA2* (−/−) tumor xenografts compared to the parental DLD-1 tumor xenografts (**Figure 3B**).

### 3.5 Loss of ATM is associated with decreased BRCA1/2 expression and inhibition of phosphorylation of MRN complex proteins NBN and RAD50

**Figure 4:**
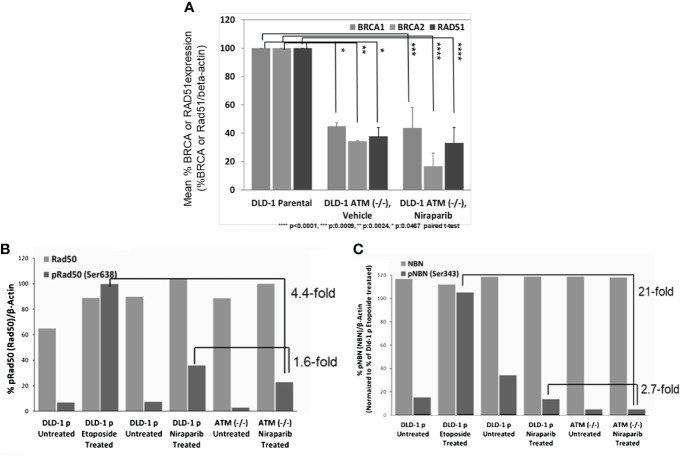
Loss of ATM is associated with low BRCA and RAD51 expression and inhibition of phosphorylation of MRN complex proteins in DLD-1 *hATM* (−/−) KO cell line. Change in BRCA1, BRCA2, and RAD51 protein levels (by WB analysis) following vehicle and niraparib treatment (50 mg/kg PO, QD 28 days or longer) in tumors from DLD-1 *hATM* (−/−) KO cell line tumor model, n=6 mice/arm **(A)**; %pRAD50(Ser638)/Rad50 and %pNBN(Ser343)/NBN expression in DLD-1 *hATM* (−/−) KO cell line by WB analysis, compared to levels in Dld-1 parental cell line (%pRad50(Rad50)/beta-actin), (n=1) **(B)**, and (%pNBN(NBN)/beta-actin), (n=1) **(C)** (niraparib at 3 µM and etoposide at 40 µM).

We further explored the impact of ATM loss on the downstream signaling of HR-mediated DNA repair by assessing if ATM loss was associated with the downregulation of BRCA1/2 expression. Towards this end, we compared BRCA1 and BRCA2 protein levels in DLD-1 *hATM* (−/−) cells to the level found in the parental DLD-1 cell line and observed that homozygous loss of ATM led to a significant downregulation of both BRCA1 and BRCA2 protein expression (**Figure 4A**). Additionally, we found that ATM loss was associated with inhibition of phosphorylation of two critical MRN complex proteins, namely, NBN and RAD50 (**Figure 4B**). Niraparib treatment induced a ~3-fold decrease in phosphorylation of NBN(Ser343) and a ~ 2-fold decrease in pRAD50(Ser638) levels in DLD-1 *hATM* (−/−) KO cell line compared to the DLD-1 parental cell line. A more robust effect was observed upon treatment with etoposide, which induced a >20-fold decrease in pNBN(Ser343) and a >4-fold decrease in pRAD50(Ser638) levels in DLD-1 *hATM* HOMO KO cell line (**Figures 4B, C**; **Supplementary Figure S5**).

In summary, our data suggest that ATM loss leads to increased sensitivity to niraparib due to the downregulation of BRCA1 and BRCA2 expression. The resulting effect of downregulation BRCA1/2 expression leads to defective DNA damage repair processes and subsequently to increased sensitivity to PARP inhibition.

### 3.6 ATM deficiency is associated with increased sensitivity to PARP inhibition in NSCLC PDX models

**Figure 5:**
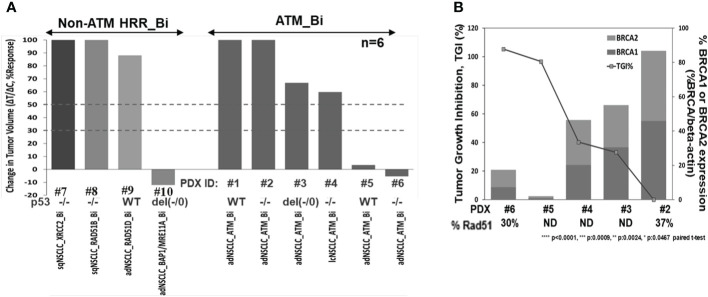
Niraparib monotherapy in vivo activity summary in HRR mutant NSCLC PDX models **(A)**, correlation analysis of tumor growth inhibition (TGI%) and downregulation of BRCA1/2 expression in the indicated ATM (−/−) PDX models; TGI%: [1−(ΔT/ΔC)]×100, following niraparib monotherapy at 50 mg/kg PO, QD for 28 days or longer, and baseline BRCA1 and 2 protein expression profiling by WB analysis; %Rad51 expression in PDX samples by WB analysis, compared to levels in Dld-1 parental cell line (%Rad51/beta-actin) **(B)**.

To investigate niraparib’s activity in vivo, we evaluated niraparib monotherapy activity in a panel of 10 NSCLC PDX models, six of which contained deleterious biallelic ATM mutations and four with non-ATM, non-BRCA HRR deleterious biallelic mutations. Niraparib monotherapy treatment caused significant tumor growth inhibition (>90% TGI) in two out of six PDX models with ATM biallelic mutations (**Figure 5A**). Additionally, the two ATM (−/−) PDX models with the greatest sensitivity to niraparib monotherapy (PDX#5 and PDX#6), expressed the lowest levels of BRCA1 and BRCA2 (**Figure 5B**). Although PDX#6 expressed slightly higher levels of BRCA1 and BRCA2, these differences do not appear to significantly impact niraparib sensitivity. Niraparib treatment had a moderate effect (TGI 30%–45%) on two of the ATM (−/−) and no effect on the remaining two ATM (−/−) models. Interestingly, the highest degree of tumor growth inhibition was observed in a PDX tumor model that contained biallelic mutations in two different HRR genes, namely, BAP1 and MRE11A (>100% TGI, **Figure 5A**). Niraparib treatment had no effect on the growth of the three BRCA^WT^, ATM^WT^ NSCLC PDX models that contained a biallelic deleterious mutation in either RAD51B, RAD51D, or XRCC2 (0%–20% TGI, **Figure 5A**). Western blot analysis confirmed that the two ATM biallelic mutant NSCLC PDX models with the highest degree of sensitivity to niraparib, i.e., LXFA 2155 (PDX#5) and LXFA 2184 (PDX#6), had no detectable ATM protein expression. In comparison, the two ATM monoallelic mutant PDX models (GXA3005 and GXA 3011) expressed detectable levels of ATM protein, albeit at relatively low levels compared to the ATM.WT DLD-1 parental, control cell line (**Supplementary Figure S4**).

### 3.7 Niraparib sensitivity in ATM (−/−) NSCLC PDX models is associated with low BRCA1 and BRCA2 expression levels

The observation that *hATM* KO in DLD-1 cells caused a decrease in BRCA1 and BRCA2 expression levels led us to examine BRCA1 and BRCA2 protein expression in the different NSCLC PDX models. As shown in **Figure 5B**, an inverse relationship was observed between BRCA1/2 expression levels and niraparib sensitivity in the five ATM biallelic mutant PDX models that were examined. Notably, the two PDX models with highest degree of niraparib response (TGI >90%) showed the lowest levels of both BRCA1 and BRCA2 proteins. In comparison, the two ATM (−/−) PDX models with moderate sensitivity to niraparib (30%–40% TGI) expressed moderate levels of BRCA1/2, and the one niraparib-insensitive ATM (−/−) PDX model that was tested had the highest level of BRCA1/2 expression. Because all ATM (−/−) NSCLC PDX models expressed low levels of RAD51, RAD51 expression levels does not appear to predict niraparib sensitivity in the PDX models examined.

Additionally, although two out of the three highly responsive ATM biallelic mutant models had deleterious mutations in p53 gene, the third model contained two copies of WT p53, suggesting that p53 status does not influence the level of sensitivity to niraparib (**Figure 5A**; **Supplementary Table S3**).

## 4 Discussion

Niraparib has demonstrated hierarchical efficacy in patients with ovarian cancer that was dependent upon the BRCA1/2 mutational and HRD statuses of their tumors. More specifically, patients whose tumors contained a germline mutation in either BRCA1 or BRCA2 (gBRCA) experienced a longer period of progression-free survival compared to patients whose tumors contained a somatic BRCA1/2 mutation (sBRCA), while patients who had BRCA1/2 wild-type, HRD-positive (HRD score >42) tumors had the shortest period of progression-free survival (19, 48). The PARP inhibitor olaparib demonstrated a similar hierarchical sensitivity in ovarian cancer patients (49). The differential benefit that ovarian cancer patients derived from niraparib monotherapy, coupled with the observation that niraparib treatment caused tumor regressions in two NSCLC patients (42), compelled us to compare niraparib activity in isogenic cell lines lacking either BRCA2 or ATM and to assess niraparib efficacy in a panel of NSCLC PDX models containing biallelic mutations in ATM and other HRR genes.

Our studies demonstrated that the homozygous loss of *hATM* in the DLD-1 cell line significantly increased the sensitivity of DLD-1 cells to niraparib by 80-fold compared to the ATM-proficient parental DLD-1 cell line. A comparison of the effect that niraparib had on the growth of DLD-1 *hBRCA2* (−/−) KO, DLD-1 *hATM* (−/−), and DLD-1 *hATM* (+/−) cell lines revealed a positive correlation between in vitro and in vivo responses to niraparib, with a consistent rank order of sensitivity among the different cell lines. The DLD-1 *hBRCA2* (−/−) KO cell line demonstrated the highest sensitivity, followed by the *hATM* (−/−) KO cell line, and the *hATM* (+/−) KO cell line being the least sensitive. Mechanistically, the investigation revealed that ATM deficiency led to a significant downregulation of both BRCA1 and BRCA2 proteins in the isogenic cell lines, resulting in impaired HRR functionality. Downstream effects also included defective, RAD51 foci, γH2AX foci and phosphorylation of MRN complex proteins NBN and RAD50, further highlighting the intricate role of ATM in DNA damage response signaling. Interestingly, the DLD-1 *hBRCA2* (−/−) cell line expressed a lower level of RAD51 than DLD-1 *hATM* (−/−) cell line, which is consistent with the observation that the DLD-1 *hBRCA2* (−/−) cell line is more sensitive to niraparib than the isogenic DLD-1 *hATM* (−/−) cell line. While additional isogenic *hATM* (−/−) and *hBRCA2* (−/−) cell lines would need to be examined to confirm this observation, the data are consistent with the clinical data showing that patients with BRCA mutant ovarian cancer have longer periods of progression-free survival compared to patients with BRCA WT, HRD+ tumors.

While our study provides valuable insights into the relationship between ATM and DNA repair pathways, it is important to acknowledge the limitations of our study in fully elucidating the mechanistic details of ATM’s regulation on BRCA/RAD51 proteins and on MRN phosphorylation. To address these gaps, we recommend conducting in-depth mechanistic studies using molecular techniques such as chromatin immunoprecipitation (ChIP) assays or gene expression analysis. These approaches would allow for the investigation of the direct or indirect regulatory roles of ATM on BRCA/RAD51 proteins or MRN phosphorylation, providing deeper insights into the underlying molecular mechanisms. Moreover, genetic and proteomic analyses represent valuable avenues for identifying potential downstream targets or pathways impacted by ATM activity. By elucidating ATM’s broader regulatory network in DNA repair processes, these analyses could uncover novel therapeutic targets and pathways for cancer treatment. In addition, the potential use of pharmacological inhibitors or activators targeting ATM or its downstream effectors holds promise for modulating DNA repair pathways. Such approaches could offer valuable insights into the development of therapeutic strategies aimed at enhancing DNA repair efficiency or sensitizing cancer cells to DNA-damaging agents. By pursuing these approaches, future studies can further advance our understanding of ATM-mediated DNA repair mechanisms.

It is noteworthy that the fold difference between wild type and *hBRCA2* (−/−) or *hATM*(−/−) for survival upon niraparib treatment in vitro (80-133X) is significantly higher than that for the mechanistic readouts, such as RAD51 and γH2AX foci in vitro (×2–4) and RAD51 and gH2AX protein levels in vivo. The discrepancies between the functional and mechanistic studies may be attributed to several factors. For example, although RAD51 and γH2AX foci provide valuable insights into the homologous recombination activity, they do not capture all the complexities of DNA repair dynamics in the context of PARPi sensitivity. It is possible that ATM loss could impact other DNA repair pathways such as NHEJ or MMEJ. Accordingly, the impact that ATM loss has on DDR pathways other than HR should also be examined in future studies. The fact that the in vivo studies were collected at the end of the studies, which may have led to a loss of signal intensity over time, could also explain the differences between the functional and mechanistic studies utilized in this paper. Additionally, the lower fold difference observed for the mechanistic readouts in the in vivo samples could be influenced by the duration of treatment and the kinetics of the DNA repair process in the tumor microenvironment. Therefore, samples may need to be collected at earlier time points during the in vivo studies to capture the dynamics of DNA repair mechanisms and treatment response accurately.

Additionally, the fact that we observed a strong correlation between niraparib’s in vitro and in vivo efficacy in the DLD-1 isogenic cell lines provided strong support for the validity and translatability of our findings. Furthermore, the demonstration that one-third of ATM mutant NSCLC PDX models are highly sensitive to PARP inhibition provides strong support for the clinical relevancy of our findings. Our study indeed acknowledges the presence of two non-responders within the ATM mutant cohort, which warrants thorough investigation. Despite harboring ATM mutations, these PDX models did not show a significant response to niraparib treatment. Future experimental testing and deeper genomic analysis of these non-responders may uncover additional genetic alterations or signaling pathways that confer resistance to niraparib. This could further help to enhance our understanding of resistance mechanisms and identify potential therapeutic targets to overcome resistance to niraparib in ATM mutant PDX models. Regrettably, we did not have access to additional ATM (−/−) NSCLC PDX models. Interestingly, the NSCLC PDX model with the highest degree of sensitivity to Niraparib contained biallelic mutations in two distinct HRR genes, namely, MRE11 and BAP1. While it is interesting to speculate that loss of multiple HRR genes might lead to hypersensitivity to PARP inhibition, the fact that HRR mutations are for the most part mutually exclusive suggests that such hypersensitivity is likely confined to a limited subset of patients. Alternatively, it is possible that the high degree of sensitivity to niraparib that was observed in our studies could be attributed to the loss of either BAP1 or MRE11 alone. Regrettably, we were unable to identify any NSCLC PDX models with BAP1 (−/−) or MRE11 (−/−) loss alone to further investigate this hypothesis.

Notably, an inverse correlation was identified between niraparib sensitivity and BRCA1/2 expression levels in ATM (−/−) NSCLC PDX models. This is a potentially important observation because only one-third of ATM mutant PDX models demonstrated significant sensitivity to niraparib treatment. More specifically, this observation suggests that ATM mutational status alone does not sufficiently identify NSCLC patients who are most likely to benefit from PARPi therapy. Therefore, the inclusion of low BRCA expression as a second biomarker (ATM−/−; low BRCA) should be strongly considered.

While our study provides a compelling rationale for exploring PARP inhibitors as targeted therapy in ATM-deficient tumors, further investigation into the functional consequences of various ATM mutations is warranted. Additionally, the exploration of drug combination therapeutic approaches holds promise in maximizing niraparib efficacy in the clinical setting. Notably, one promising combination involves combining PARP inhibitors with VEGF TKIs. Previous evidence presented by Kaplan et al. (50) demonstrated that the VEGFR TKI cediranib suppresses HR-mediated DNA repair through the downregulation of BRCA1/2 and RAD51. Importantly, this cediranib-mediated decrease in BRCA expression was associated with increased sensitivity of tumor xenografts to the PARPi olaparib. Importantly, this effect was observed in the tumor tissue but not in mouse bone marrow, creating a therapeutic window for combining cediranib with a PARPi in cancer therapy.

## Data availability statement

The original contributions presented in the study are included in the article/**Supplementary Material**. Further inquiries can be directed to the corresponding authors.

## Ethics statement

Ethical approval was not required for the studies on humans in accordance with the local legislation and institutional requirements because only commercially available established cell lines were used. The animal study was approved by Charles River Laboratories Germany GmbH: The study was conducted according to all applicable international, national and local laws and followed the national guidelines for the Care and Use of Laboratory Animals of the Society of Laboratory Animal Science (GV-SOLAS). All animal experiment protocols were approved by the regional council Committee on the Ethics of Animal Experiments. The study was conducted in accordance with the local legislation and institutional requirements. All procedures for in vivo studies were conducted according to all applicable international, national, and local laws and followed the national guidelines for the Care and Use of Laboratory Animals of the Society of Laboratory Animal Science (GV-SOLAS). All animal experiment protocols were approved by the regional council Committee on the Ethics of Animal Experiments.

## Author contributions

AM: Conceptualization, Data curation, Formal analysis, Investigation, Methodology, Resources, Supervision, Validation, Writing – original draft, Writing – review & editing. KC: Conceptualization, Investigation, Supervision, Validation, Writing – original draft, Writing – review & editing.

## References

[1] Ashworth A Lord CJ. Synthetic lethal therapies for cancer: What’s next after PARP inhibitors? Nat Reviews: Clin Oncol. (2018) 15:564–76. doi: 10.1038/s41571-018-0055-6 29955114

[2] O’Connor MJ. Targeting the DNA damage response in cancer. Mol Cell. (2015) 60:547–60. doi: 10.1016/j.molcel.2015.10.040 26590714

[3] Leibowitz BD Dougherty BV Bell JSK Kapilivsky J Michuda J Sedgewick AJ. Validation of genomic and transcriptomic models of homologous recombination deficiency in a real-world pan-cancer cohort. BMC Cancer. (2022) 22:587. doi: 10.1186/s12885-022-09669-z 35643464 PMC9148513

[4] McCarthy-Leo C Darwiche F Tainsky MA. DNA repair mechanisms, protein interactions and therapeutic targeting of the MRN complex. Cancers (Basel). (2022) 14:5278. doi: 10.3390/cancers14215278 36358700 PMC9656488

[5] Krajewska M Fehrmann RS Schoonen PM Labib S de Vries EG Franke L. ATR inhibition preferentially targets homologous recombination-deficient tumor cells. Oncogene. (2015) 34(26):3474–81. doi: 10.1038/onc.2014.276 25174396

[6] Farmer H McCabe N Lord CJ Tutt AN Johnson DA Richardson TB. Targeting the DNA repair defect in BRCA mutant cells as a therapeutic strategy. Nature. (2005) 434(7035):917–21. doi: 10.1038/nature03445 15829967

[7] Konstantinopoulos PA Ceccaldi R Shapiro GI D’Andrea AD. Homologous recombination deficiency: Exploiting the fundamental vulnerability of ovarian cancer. Cancer Discov. (2015) 5(11):1137–54. doi: 10.1158/2159-8290.CD-15-0714 PMC4631624 26463832

[8] Lord CJ Ashworth A. BRCAness revisited. Nat Reviews: Cancer. (2016) 16:111–20. doi: 10.1038/nrc.2015.21 26775620

[9] Zhu H Wei M Xu J Hua J Liang C Meng Q. PARP inhibitors in pancreatic cancer: molecular mechanisms and clinical applications. Mol Cancer. (2020) 19:49. doi: 10.1186/s12943-020-01167-9 32122376 PMC7053129

[10] Carreira S Porta N Arce-Gallego S Seed G Llop-Guevara A Bianchini D. Biomarkers associating with PARP inhibitor benefit in prostate cancer in the TOPARP-B trial. Cancer Discovery. (2021) 11:2812–27. doi: 10.1158/2159-8290.CD-21-0007 PMC9414325 34045297

[11] Yoshida K Miki Y. Role of BRCA1 and BRCA2 as regulators of DNA repair, transcription, and cell cycle in response to DNA damage. Cancer Sci. (2004) 95(11):866–71. doi: 10.1111/j.1349-7006.2004.tb02195.x PMC11159131 15546503

[12] Brandsma I Fleuren EDG Williamson CT Lord CJ. Directing the use of DDR kinase inhibitors in cancer treatment. Expert Opin Investig Drugs. (2017) 26(12):1341–1355. doi: 10.1080/13543784.2017.1389895 PMC6157710 28984489

[13] Lee JH Paull TT. Cellular functions of the protein kinase ATM and their relevance to human disease. Nat Rev Mol Cell Biol. (2021) 22:796–814. doi: 10.1038/s41580-021-00394-2 34429537

[14] Smith HL Southgate H Tweddle DA Curtin NJ. DNA damage checkpoint kinases in cancer. Expert Rev Mol Med. (2020) 22:e2. doi: 10.1017/erm.2020.3 32508294

[15] Xu Y Ashley T Brainerd EE Bronson RT Meyn MS Baltimore D. Targeted disruption of ATM leads to growth retardation, chromosomal fragmentation during meiosis, immune defects, and thymic lymphoma. Genes Dev. (1996) 10:2411–22. doi: 10.1101/gad.10.19.2411 8843194

[16] Mason JM Chan YL Weichselbaum RW Bishop DK. Non-enzymatic roles of human RAD51 at stalled replication forks. Nat Commun. (2019) 10:4410. doi: 10.1038/s41467-019-12297-0 31562309 PMC6764946

[17] Bryant H Schultz N Thomas H Parker KM Flower D Lopez E. Specific killing of BRCA2-deficient tumours with inhibitors of poly(ADP-ribose) polymerase. Nature. (2005) 434(7035):913–7. doi: 10.1038/nature03443 15829966

[18] Kim G Ison G McKee AE Zhang H Tang S Gwise T. FDA approval summary: olaparib monotherapy in patients with deleterious germline BRCA-mutated advanced ovarian cancer treated with three or more lines of chemotherapy. Clin Cancer Res. (2015) 21:4257–61. doi: 10.1158/1078-0432.CCR-15-0887 26187614

[19] Mirza MR Monk BJ Herrstedt J Oza AM Mahner S Redondo A. for the ENGOT-OV16/NOVA investigators. Niraparib maintenance therapy in platinum-sensitive, recurrent ovarian cancer. N Engl J Med. (2016) 375(22):2154–64. doi: 10.1056/nejmoa1611310 27717299

[20] Oza AM Tinker AV Oaknin A Shapira-Frommer R McNeish IA Swisher EM. Antitumor activity and safety of the PARP inhibitor rucaparib in patients with high-grade ovarian carcinoma and a germline or somatic BRCA1 or BRCA2 mutation: Integrated analysis of data from Study 10 and ARIEL2. Gynecol Oncol. (2017) 147:267–75. doi: 10.1016/j.ygyno.2017.08.022 28882436

[21] Marshall CH Sokolova AO McNatty AL Cheng HH Eisenberger MA Bryce AH. Differential response to olaparib treatment among men with metastatic castration-resistant prostate cancer harboring BRCA1 or BRCA2 versus ATM mutations. Eur Urol. (2019) 76:452–8. doi: 10.1016/j.eururo.2019.02.002 PMC6703974 30797618

[22] Franza A Claps M Procopio G. PARP inhibitors and metastatic castration-resistant prostate cancer: Future directions and pitfalls. Trans Oncol. (2022) 15:101263. doi: 10.1016/j.tranon.2021.101263 PMC8591349 34763215

[23] Mateo J Carreira S Sandhu S Miranda S Mossop H Perez-Lopez R. DNA-repair defects and olaparib in metastatic prostate cancer. New Engl J Med. (2015) 373:1697–708. doi: 10.1056/NEJMoa1506859 PMC5228595 26510020

[24] Ison G Howie LJ Amiri-Kordestani L Zhang L Tang S Sridhara R. FDA approval summary: niraparib for the maintenance treatment of patients with recurrent ovarian cancer in response to platinum-based chemotherapy. Clin Cancer Res. (2018) 24:4066–71. doi: 10.1158/1078-0432.CCR-18-0042 29650751

[25] Lord CJ Ashworth A. PARP inhibitors: The first synthetic lethal targeted therapy. Science. (2017) 355:1152–8. doi: 10.1126/science.aam7344 PMC6175050 28302823

[26] Coleman RL Oza AM Lorusso D Aghajanian C Oaknin A Dean A. ARIEL3 investigators. Rucaparib maintenance treatment for recurrent ovarian carcinoma after response to platinum therapy (ARIEL3): a randomised, double-blind, placebo-controlled, phase 3 trial. *Lancet* . (2018) 390(10106):1949–61. doi: 10.1016/S0140-6736(17)32440-6 PMC5901715 28916367

[27] Williamson CT Muzik H Turhan AG Zamò A O’Connor MJ Bebb DG. ATM deficiency sensitizes mantle cell lymphoma cells to poly (ADP-ribose) polymerase-1 inhibitors. Mol Cancer Ther. (2010) 9:347–57. doi: 10.1158/1535-7163.MCT-09-0872 PMC3729269 20124459

[28] Villaruz LC Jones H Dacic S Abberbock S Kurland BF Stabile LP. ATM protein is deficient in over 40% of lung adenocarcinomas. Oncotarget. (2016) 7(36):57714–25. doi: 10.18632/oncotarget.9757 PMC5295384 27259260

[29] Schmitt A Knittel G Welcker D Yang T George J Nowak M. ATM deficiency is associated with sensitivity to PARP1- and ATR inhibitors in lung adenocarcinoma. Cancer Res. (2017) 77:3040–56. doi: 10.1158/0008-5472.CAN-16-3398 28363999

[30] Jette NR Kumar M Radhamani S Arthur G Goutam S Yip S. ATM-deficient cancers provide new opportunities for precision oncology. Cancers (Basel). (2020) 12:687. doi: 10.3390/cancers12030687 32183301 PMC7140103

[31] Mateo J Porta N Bianchini D McGovern U Elliott T Jones R. Olaparib in patients with metastatic castration-resistant prostate cancer with DNA repair gene aberrations (TOPARP-B): a multicentre, open-label, randomised, phase 2 trial. Lancet Oncol. (2020) 21(1):162–74. doi: 10.1016/S1470-2045(19)30684-9 PMC6941219 31806540

[32] The Cancer Genome Atlas Research Network. Comprehensive molecular profiling of lung adenocarcinoma. Nature. (2014) 511:543–50. doi: 10.1038/nature13385 PMC4231481 25079552

[33] The TCGA Research Network. Available at: https://www.cancer.gov/tcga (Accessed September, 2019).

[34] Perkhofer L Schmitt A Romero Carrasco MC Ihle M Hampp S Ruess DA. ATM deficiency generating genomic instability sensitizes pancreatic ductal adenocarcinoma cells to therapy-induced DNA damage. Cancer Res. (2017), 5576–90. doi: 10.1158/0008-5472.CAN-17-0634 28790064

[35] Malhotra J Jabbour SK Pine S. Impact of surveillance frequency on survival in non-small cell lung cancer (NSCLC) survivors. Transl Lung Cancer Res. (2019) 8:S347–50. doi: 10.21037/tlcr PMC6987361 32038912

[36] Sung H Ferlay J Siegel RL Laversanne M Soerjomataram I Jemal A. Global cancer statistics 2020: GLOBOCAN estimates of incidence and mortality worldwide for 36 cancers in 185 countries. CA Cancer J Clin. (2021) 71(3):209–49. doi: 10.3322/caac.21660 33538338

[37] Mak RH Hermann G Aerts HJ Baldini EH Chen AB Kozono D. Outcomes by EGFR, KRAS, and ALK genotype after combined modality therapy for locally advanced non-small-cell lung cancer. JCO Precis Oncol. (2018) 2:1–18. doi: 10.1200/PO.17.00219 35135121

[38] Huo G Liu W Chen P. Inhibitors of PD-1 in non-small cell lung cancer: A meta-analysis of clinical and molecular features. Front Immunol. (2022) 13:875093. doi: 10.3389/fimmu.2022.875093 35479081 PMC9037098

[39] Trakarnphornsombat W Kimura H. Live-cell tracking of γ-H2AX kinetics reveals the distinct modes of ATM and DNA-PK in the immediate response to DNA damage. J Cell Sci. (2023) 136:jcs260698. doi: 10.1242/jcs.260698 36999484 PMC10163350

[40] Vokes NI Galan Cobo A Fernandez-Chas M Molkentine D Treviño S 3rd Druker V. ATM mutations associate with distinct co-mutational patterns and therapeutic vulnerabilities in NSCLC. Clin Cancer Res. (2023) 29:4958–72. doi: 10.1158/1078-0432.CCR-23-1122 PMC10690143 37733794

[41] Chi KN Rathkopf D Smith MR Efstathiou E Attard G Olmos D. Niraparib and abiraterone acetate for metastatic castration-resistant prostate cancer. J Clin Oncol. (2023) 41:3339–51. doi: 10.1200/JCO.22.01649 PMC10431499 36952634

[42] Sandhu SK Schelman WR Wilding G Moreno V Baird RD Miranda S. The poly (ADP-ribose) polymerase inhibitor niraparib (MK4827) in BRCA mutation carriers and patients with sporadic cancer: a phase 1 dose-escalation trial. Lancet Oncol. (2013) 14:882–92. doi: 10.1016/S1470-2045(13)70240-7 23810788

[43] Kubota E Williamson CT Ye R Elegbede A Peterson L Lees-Miller SP. Low ATM protein expression and depletion of p53 correlates with olaparib sensitivity in gastric cancer cell lines. Cell Cycle. (2014) 13:2129–37. doi: 10.4161/cc.29212 PMC4111704 24841718

[44] Wang C Jette N Moussienko D Bebb DG Lees-Miller SP. ATM-deficient colorectal cancer cells are sensitive to the PARP inhibitor olaparib. Trans Oncol. (2017) 10:190–6. doi: 10.1016/j.tranon.2017.01.007 PMC5299208 28182994

[45] Shen Y Rehman FL Feng Y Boshuizen J Bajrami I Elliott R. BMN 673, a novel and highly potent PARP1/2 inhibitor for the treatment of human cancers with DNA repair deficiency. Clin Cancer Res. (2013) 19:5003–15. doi: 10.1158/1078-0432.CCR-13-1391 PMC6485449 23881923

[46] Weston VJ Oldreive CE Skowronska A Oscier DG Pratt G Dyer MJ. The PARP inhibitor olaparib induces significant killing of ATM-deficient lymphoid tumor cells in vitro and in vivo. Blood. (2010) 116:4578–87. doi: 10.1182/blood-2010-01-265769 20739657

[47] Gilardini Montani MS Prodosmo A Stagni V Merli D Monteonofrio L Gatti V. ATM-depletion in breast cancer cells confers sensitivity to PARP inhibition. J Exp Clin Cancer Res. (2013) 32:95. doi: 10.1186/1756-9966-32-95 24252502 PMC4176289

[48] Maiorano MFP Maiorano BA Biancofiore A Cormio G Maiello E. Niraparib and advanced ovarian cancer: A beacon in the non-BRCA mutated setting. Pharm (Basel). (2023) 16:1261. doi: 10.3390/ph16091261 PMC10536506 37765068

[49] Ledermann JA Pujade-Lauraine E. Olaparib as maintenance treatment for patients with platinum-sensitive relapsed ovarian cancer. Ther Adv Med Oncol. (2019) 11:1758835919849753. doi: 10.1177/1758835919849753 31205507 PMC6535754

[50] Kaplan AR Gueble SE Liu Y Oeck S Kim H Yun Z. Cediranib suppresses homology-directed DNA repair through down-regulation of BRCA1/2 and RAD51. Sci Transl Med. (2019) 11:eaav4508. doi: 10.1126/scitranslmed.aav4508 31092693 PMC6626544

