# Supplementary material for: PARP inhibitor synthetic lethality in ATM biallelic mutant cancer cell lines is associated with BRCA1/2 and RAD51 downregulation
Source: Front Oncol. 2024 May 14;14:1380633. doi: 10.3389/fonc.2024.1380633 (PMC11131418; doi:10.3389/fonc.2024.1380633)

**Supplemental Tables and Figures**

**Table S1. sgRNA Sequences for Generation of *hATM*, and *hBRCA2* Heterozygous (+/-), and Homozygous (-/-) KO Isogenic Cell Lines.**

**Table S2.** Summary of Niraparib Sensitivity in HeLa hATM -/-, and hBRCA2 -/- KO cell lines from CFA.

**
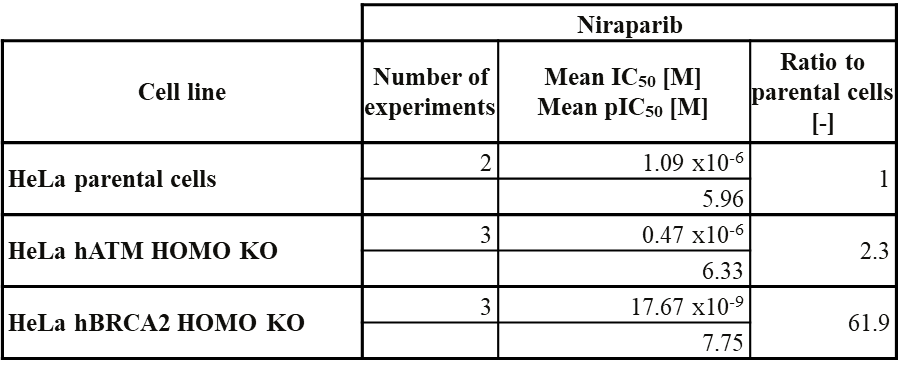
**

**Table S3. List of PDX Models, Mutation Summary and Patient Demographics.**


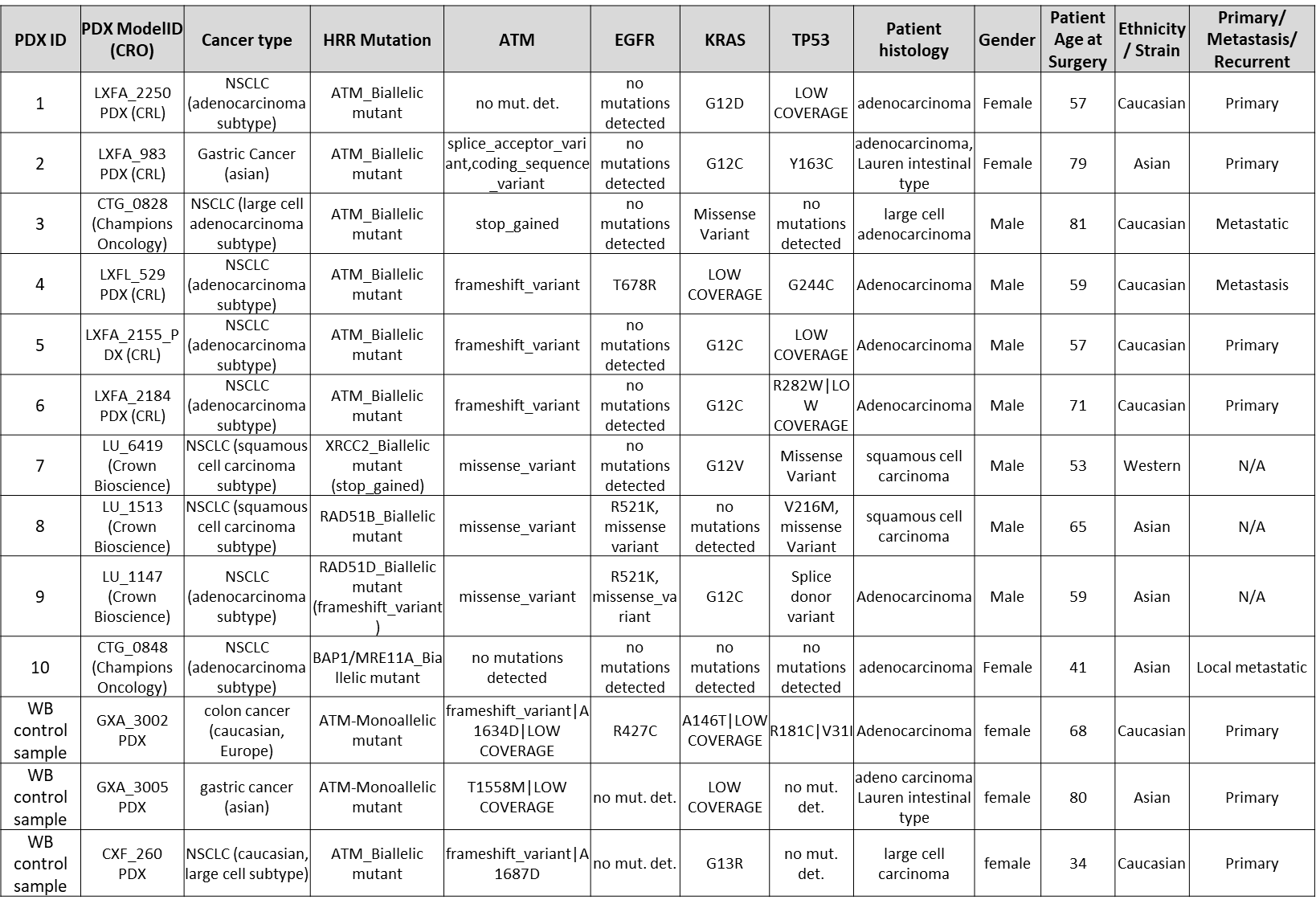


**Figure S1.** The mean foci distribution of γH2AX and RAD51 foci across 9-point dose titrations of niraparib treatment (with top dose of 10uM) in DLD-1 Parental, DLD-1 *hBRCA2* HOMO KO B4 and DLD-1 *hATM* HOMO KO at 18h, 72h, 120h, 168h, 240h.


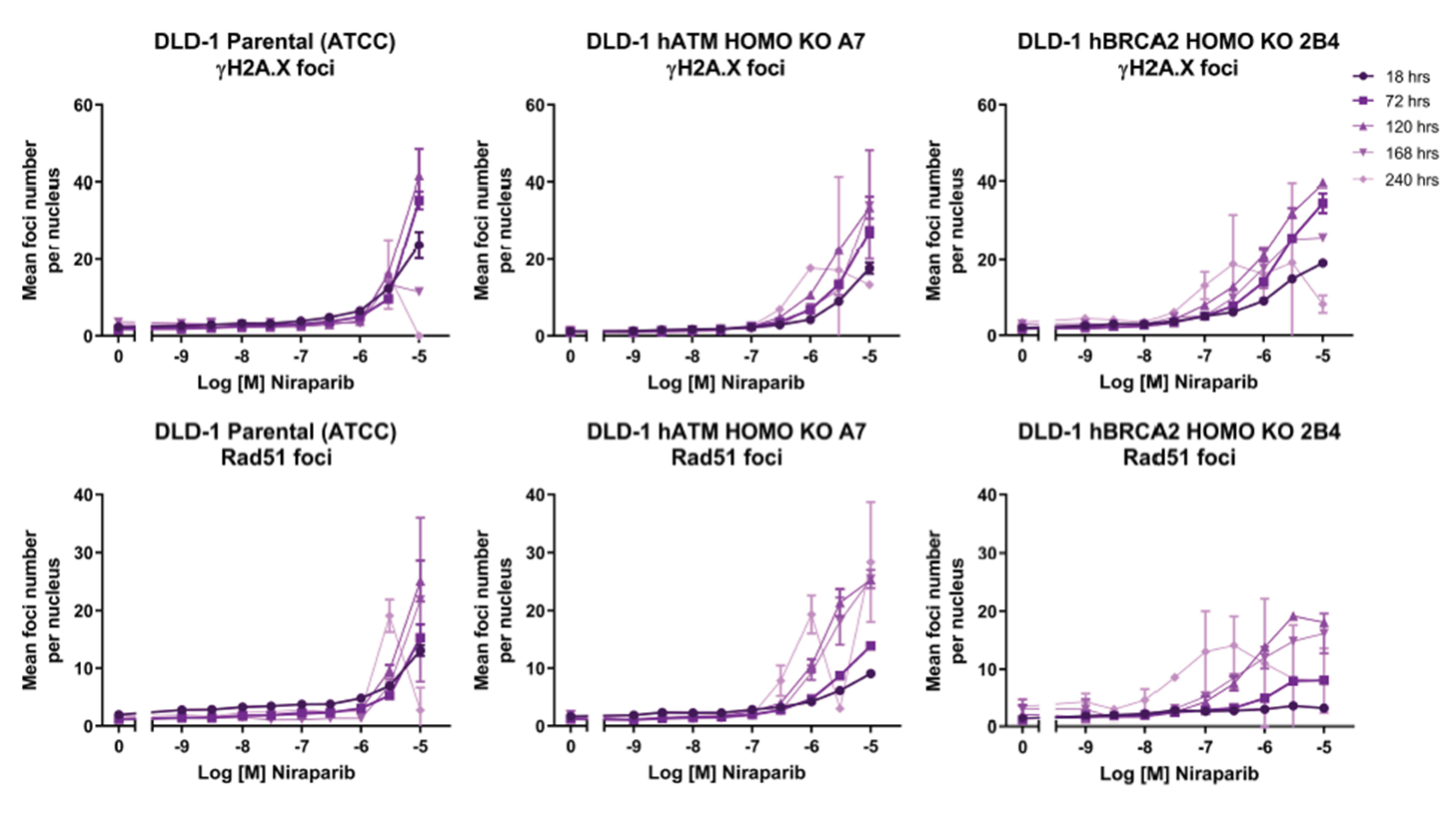


**Figure S2.** The % of positive cells (>10-foci/nucleus) distribution of γH2AX and RAD51 foci across 9-point dose titrations of niraparib treatment (with top dose of 10µM) in DLD-1 Parental, DLD-1 *hBRCA2* HOMO KO B4 and DLD-1 *hATM* HOMO KO at 18h, 72h, 120h, 168h, 240h.


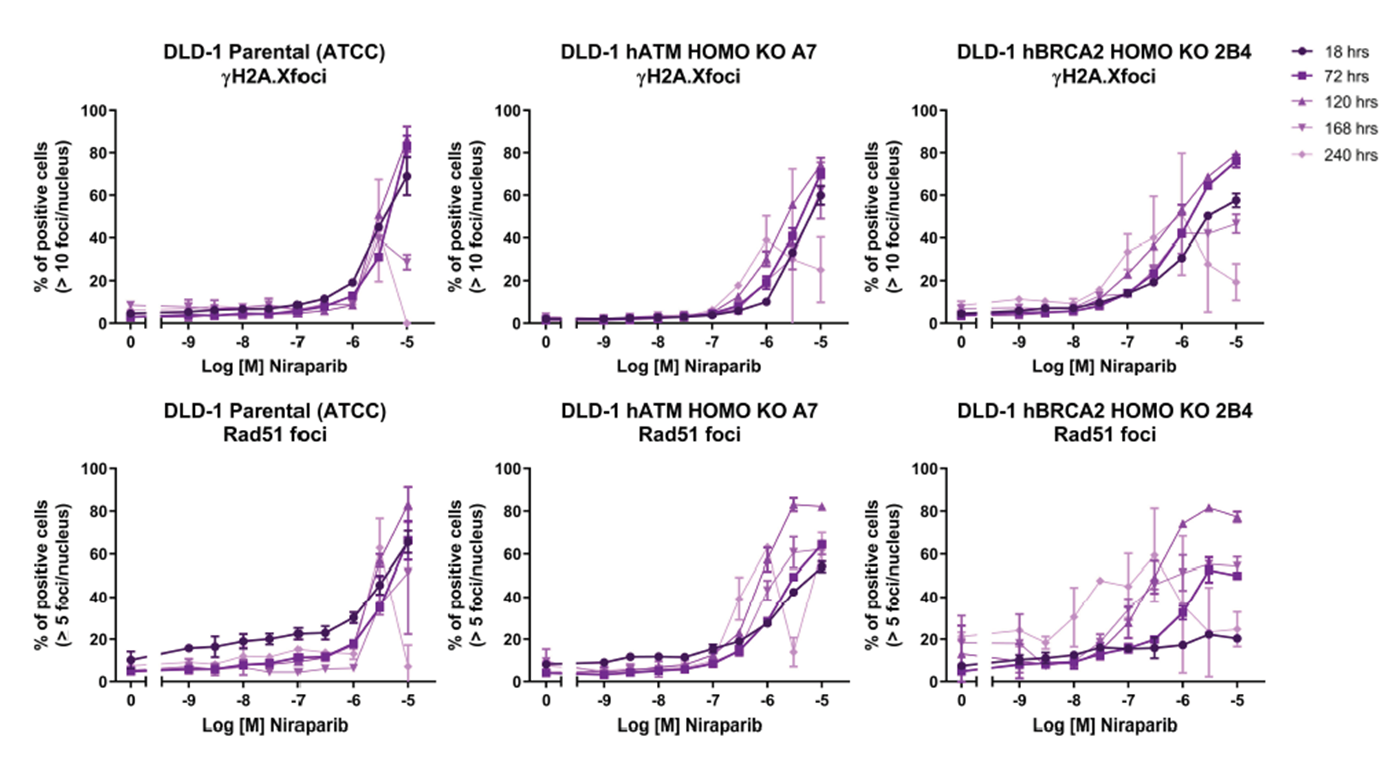


**Figure S3.** DLD-1 Parental γH2AX foci **(A)**


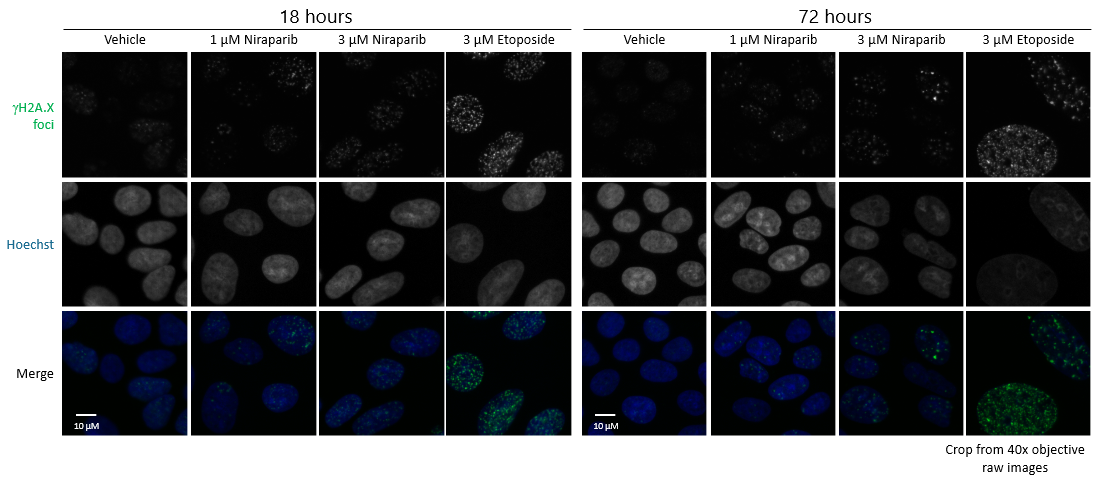


**Figure S3.** DLD-1 *hATM* (-/-) γH2AX foci **(B)**


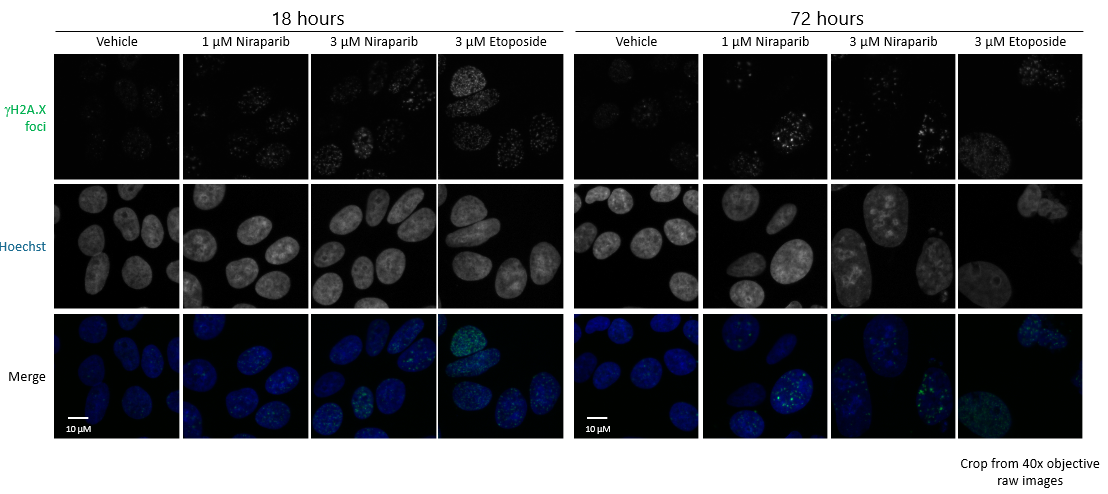


**Figure S3.** DLD-1 *hBRCA2* (-/-) B4 γH2AX foci **(C)**


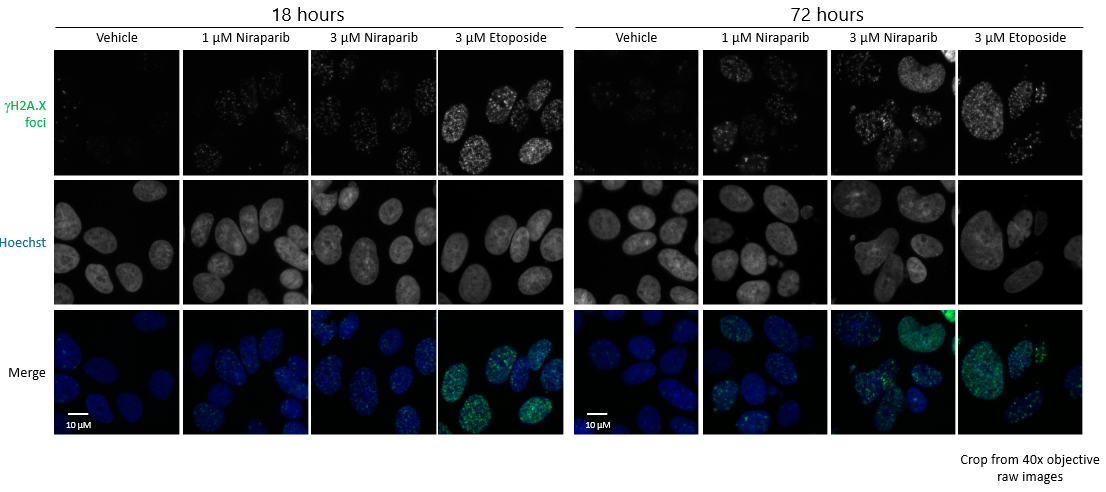


**Figure S3.** The mean foci distribution of γH2AX (in vitro) at 3µM of Etoposide treatment in DLD-1 Parental, DLD-1 *hBRCA2* HOMO KO B4 and DLD-1 *hATM* HOMO KO at 18h, and 72h, (For all results, the mean foci count and the percentage of foci positive cells (>10 γH2A.X foci/nucleus) were plotted. The average of the duplicate wells + SEM is shown. The percentage of Geminin positive nuclei was plotted) **(D).**

**
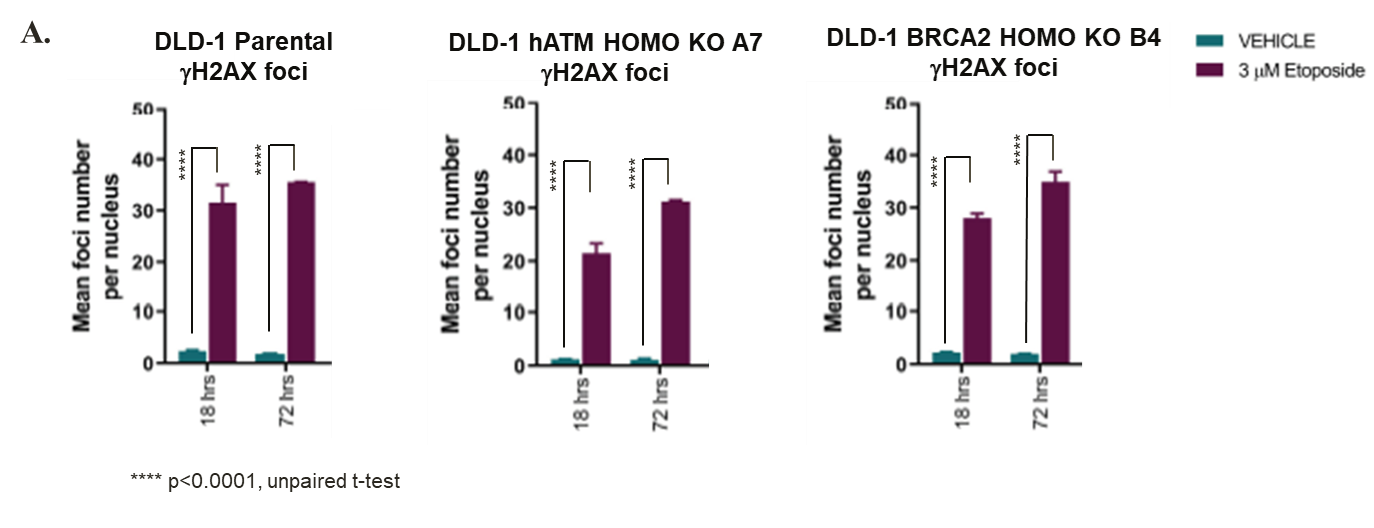
**

**Figure S3.** DLD-1 Parental RAD51 foci and Geminin **(E)**

**
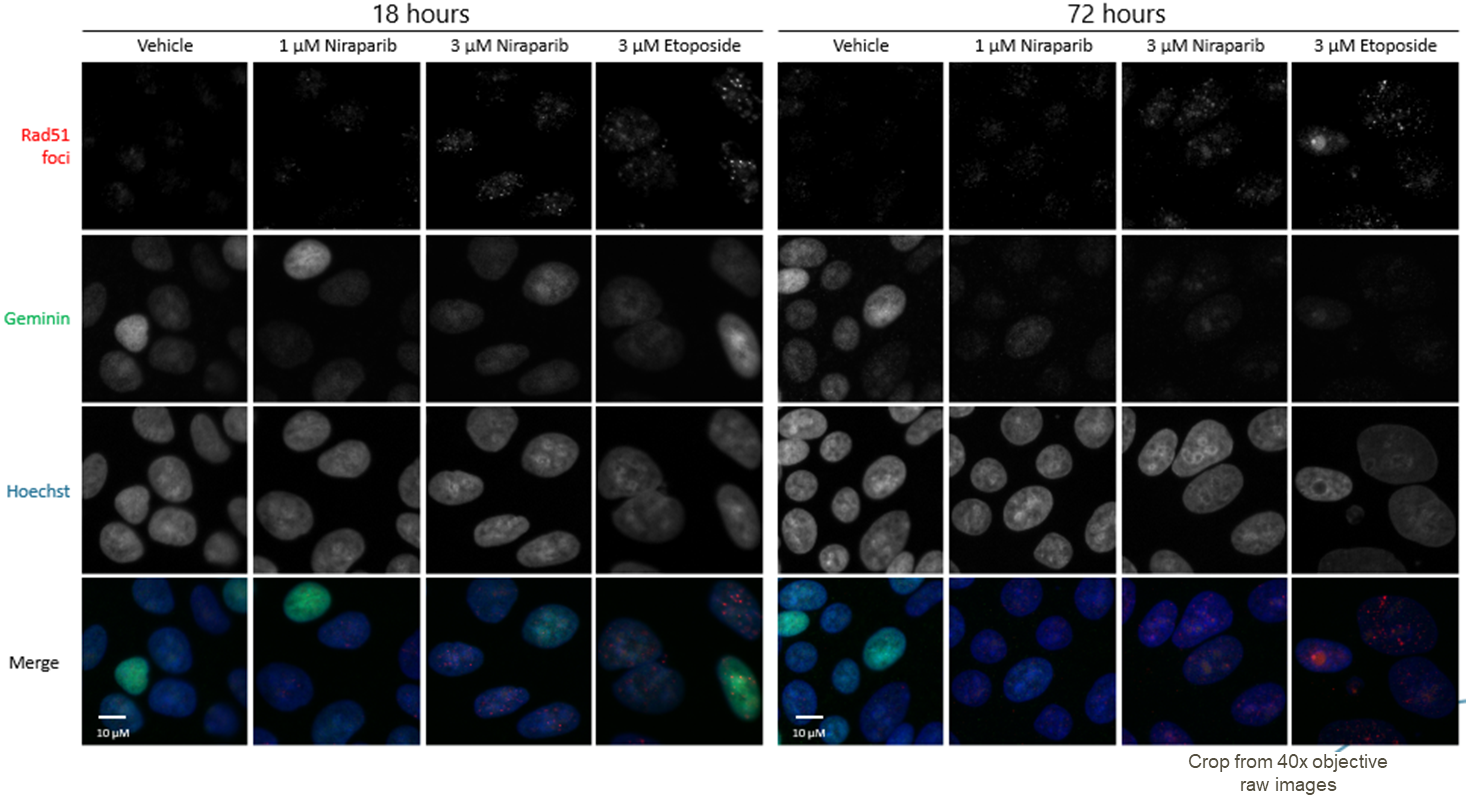
**

**Figure S3.** DLD-1 *hATM* HOMO KO A7 Rad51 foci and Geminin **(F)**


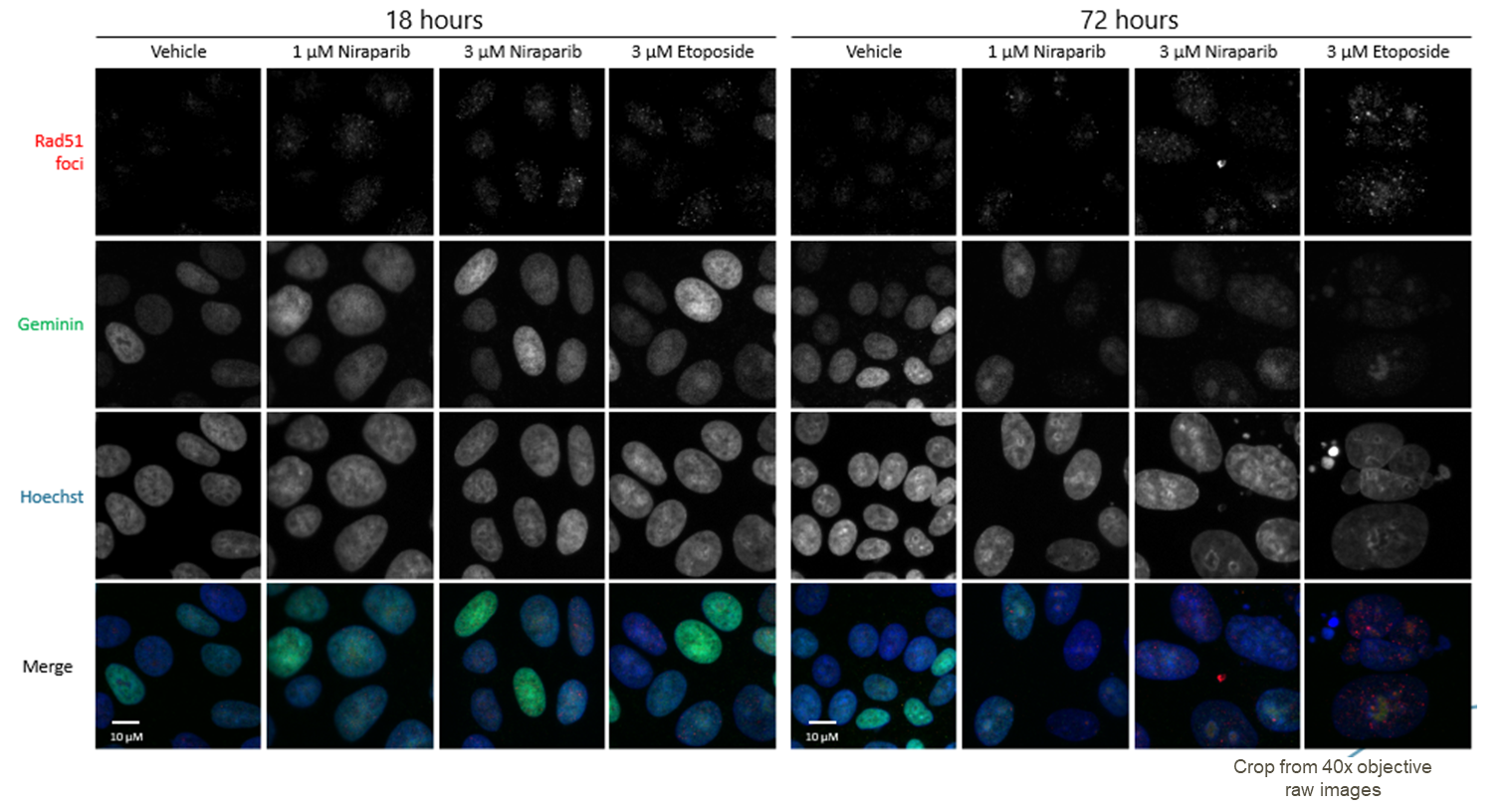


**Figure S3.** DLD-1 *hBRCA2* HOMO KO 2B4 Rad51 foci and Geminin **(G)**


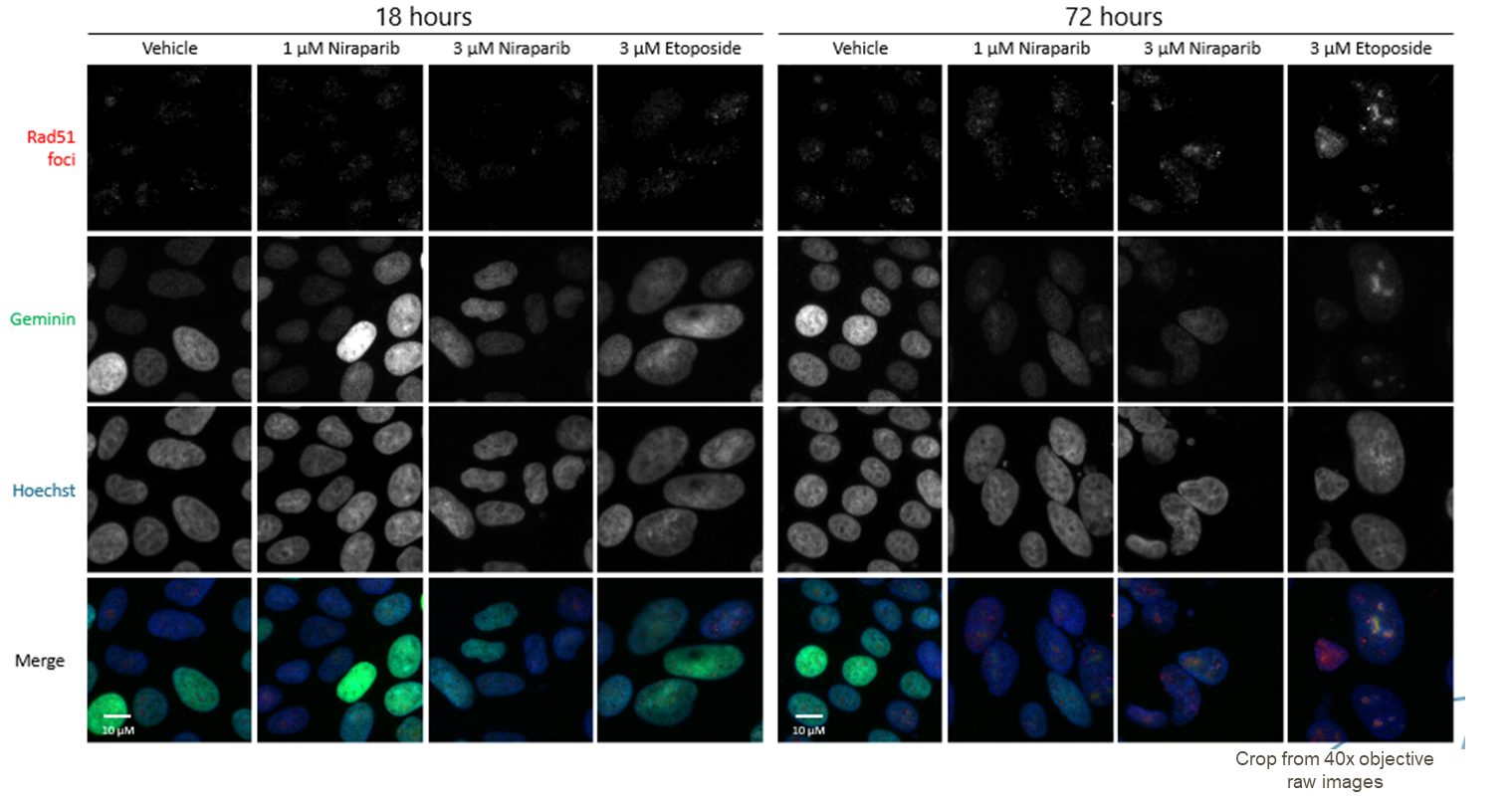


**Figure S4.** ATM Expression Profiling in ATM Biallelic Mutant PDX Models by WB Analysis


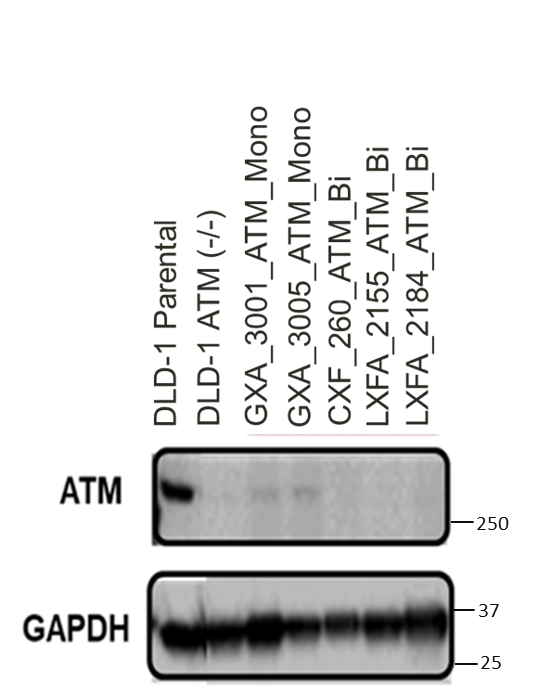


**Figure S5.** WB Images of pRAD50/RAD50 in DLD-1 Parental, and DLD-1 ATM (-/-) KO Isogenic Cell Lines.


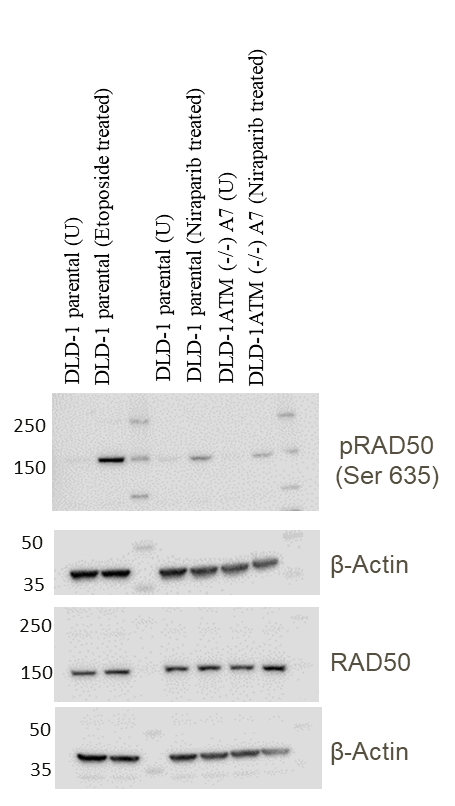

Supplement: Supplementary file 1 [file DataSheet_1.docx]
